# Synthesis and In Vitro Antitumor Activity Evaluation of Gefitinib-1,2,3-Triazole Derivatives

**DOI:** 10.3390/molecules29040837

**Published:** 2024-02-13

**Authors:** Zijun Liu, Jiancheng Liu, En Gao, Longfei Mao, Shu Hu, Sanqiang Li

**Affiliations:** 1 College of Basic Medicine and Forensic Medicine, Henan University of Science and Technology, Luoyang 471023, China; zijun01287@163.com (Z.L.); liujiancheng2017@126.com (J.L.); 2 School of Chemistry and Chemical Engineering, Henan Normal University, Xinxiang 453007, China; ge_163@126.com

**Keywords:** gefitinib, 1,2,3-triazole, antitumor drug, cell apoptosis, cell migration

## Abstract

In this study, 14 structurally novel gefitinib-1,2,3-triazole derivatives were synthesized using a click chemistry approach and characterized by ^1^H NMR, ^13^C NMR and high-resolution mass spectrometry (HRMS). Preliminary cell counting kit-8 results showed that most of the compounds exhibit excellent antitumor activity against epidermal growth factor receptor wild-type lung cancer cells NCI-H1299, A549 and NCI-H1437. Among them, **4b** and **4c** showed the most prominent inhibitory effects. The half maximal inhibitory concentration (IC_50_) values of **4b** were 4.42 ± 0.24 μM (NCI-H1299), 3.94 ± 0.01 μM (A549) and 1.56 ± 0.06 μM (NCI-1437). The IC_50_ values of **4c** were 4.60 ± 0.18 µM (NCI-H1299), 4.00 ± 0.08 μM (A549) and 3.51 ± 0.05 μM (NCI-H1437). Furthermore, our results showed that **4b** and **4c** could effectively inhibit proliferation, colony formation and cell migration in a concentration-dependent manner, as well as induce apoptosis in H1299 cells. In addition, **4b** and **4c** exerted its anti-tumor effects by inducing cell apoptosis, upregulating the expression of cleaved-caspase 3 and cleaved-PARP and downregulating the protein levels of Bcl-2. Based on these results, it is suggested that **4b** and **4c** be developed as potential new drugs for lung cancer treatment.

## 1. Introduction

**Figure 1:**
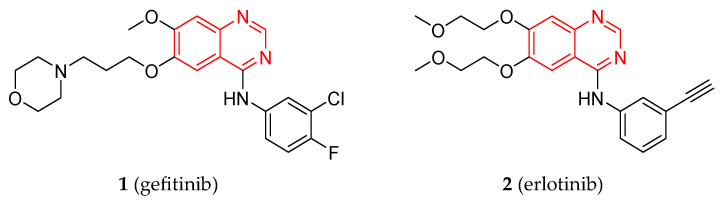
The structures of gefitinib and erlotinib. The red parts represent the parent nucleus, and the black parts represent diverse substituents.

Epidermal growth factor receptor (EGFR), encoded by the human epidermal receptor 1 (*HER1*, *ErbB1*) gene, is a cell surface receptor that belongs to the ERBB protein receptor family along with HER2/neu (ErbB2), HER3 (ErbB3) and HER4 (ErbB4). These receptor tyrosine kinase transmembrane proteins play a crucial role in regulating the proliferation, differentiation [1] and apoptosis of tumor cells. Studies have shown that inhibiting the activity and expression of tyrosine kinases can effectively suppress tumor growth and proliferation. Therefore, targeting EGFR has become a hot topic in the research of antitumor drugs. The United States Food and Drug Administration approved the first-generation EGFR tyrosine kinase inhibitors (TKIs)—gefitinib and erlotinib [2]—in 2003 and 2004, respectively. Initially approved for patients with advanced non-small cell lung cancer (NSCLC) who had not responded favorably to conventional chemotherapy, these agents demonstrated efficacy in a subset of participants during preliminary clinical trials [3,4]. In 2004, several clinical studies [5,6] showed that the sensitivity of NSCLC to erlotinib or gefitinib is associated with specific activating mutations in the EGFR gene (Figure 1). In 2006, Japanese researchers [7] conducted a phase II clinical trial, using gefitinib as a first-line treatment for 16 patients with advanced NSCLC with EGFR mutations, achieving a response rate of 75%. Subsequent large-scale phase III randomized controlled trials confirmed the superiority of EGFR-TKIs as the first-line treatment for patients with NSCLC with EGFR mutations. Compared to traditional chemotherapy, EGFR-TKIs demonstrated better progression-free survival, objective response rate, quality of life and tolerability. The use of targeted drugs has increased the median survival of patients from 4 months to over 40 months. These studies have further established the role of EGFR-TKIs for the first-line treatment of EGFR-mutant advanced NSCLC. EGFR-TKIs exhibit good inhibitory effects on EGFR-mutant lung cancer cells. However, their activity is weaker against wild-type than mutant lung cancer cells. We aimed to enhance the inhibitory activity against wild-type lung cancer cells by modifying the structure of EGFR-TKIs.

The 1,2,3-triazole is a crucial class of nitrogen-containing heterocyclic compounds, consisting of a five-membered ring constructed from three nitrogen atoms and two carbon atoms [8]. With a molecular formula of C_2_N_3_H_3_, 1,2,3-triazoles possess a unique planar and rigid structure, granting them the robust ability to intercalate with DNA. Additionally, they exhibit a large dipole moment, allowing for various non-covalent interactions with different biological targets such as hydrophobic, hydrogen bonding, van der Waals forces and dipole–dipole interactions [9]. Moreover, the structural characteristics of 1,2,3-triazoles permit them to serve as electronic equivalents of amides, esters, carboxylic acids and rigid analogs of olefins, endowing them with broad-spectrum biological activities [10]. Consequently, they are often employed as crucial molecular building blocks for the synthesis of active compounds, including antibacterial [11], antimalarial [12], antifungal [13], antiviral [14], antitubercular [15] and anticancer agents [16,17], showcasing its widespread applications in the field of medicinal chemistry.

**Figure 2:**
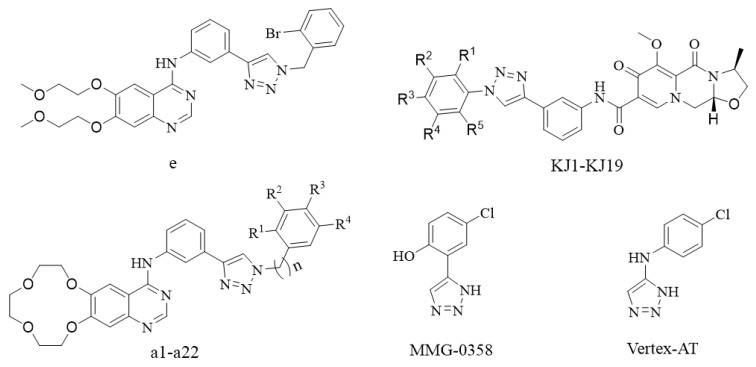
Structures of compounds with 1,2,3-triazole.

Numerous clinical drugs have demonstrated enhanced or novel biological activities through modification with 1,2,3-triazoles. For example, five erlotinib-conjugated 1,2,3-triazole derivatives were designed using a structure-based drug design strategy to explore novel IDO1 inhibitors. Among them, compound e with an o-bromobenzyl structure on the triazole ring showed the best IDO1 inhibitory activity (IC_50_ = 0.32 ± 0.07 μM) [18]. In another study, the anti-HIV drug cabotegravir was modified by introducing a 1,2,3-triazole moiety to obtain compounds KJ1–KJ19. These compounds exhibited notable inhibitory effects on the proliferation of HepG2 liver cancer cells. Compounds KJ-5 and KJ-12 showed promising inhibitory potencies in particular. Furthermore, both KJ-5 and KJ-12 were found to induce apoptosis via the mitochondrial pathway and inhibit the invasion and migration of cancer cells. [19]. By utilizing the structural features of the EGFR inhibitor erlotinib, compounds a1–a22 with erlotinib-1,2,3-triazole derivatives were obtained through click reactions [20]. These compounds exhibited excellent inhibitory activity against five NSCLC cell lines, three esophageal squamous cell carcinoma cell lines and some chemotherapy-resistant tumor cell lines. Compound a12 showed the best performance (half maximal inhibitory concentration [IC_50_] < 10 M) and demonstrated strong antiproliferative and anticloning abilities against various tumor cells. Additionally, it induced apoptosis and cell cycle arrest in tumor cells. Compound 3l, targeting EGFR, exhibited binding to EGFR protein and downregulated the expression of phosphorylated extracellular signal-regulated kinase and Ki-67, demonstrating potential as an EGFR inhibitor [21]. Moreover, compounds MMG-0358 and Vertex-AT, incorporating 1,2,3-triazole into the phenyl ring and aniline structure, were developed as indoleamine 2,3-dioxygenase-1 (IDO1) inhibitors. These compounds deeply penetrate the target protein of IDO1, forming hydrogen bonds with the ferrous ion in the heme of the target. The triazole-linked phenyl ring can penetrate a hydrophobic pocket surrounded by amino acids Cys129, Leu234 and Gly262. MMG-0358 and Vertex-AT both exhibit outstanding IDO1 inhibitory activity, with IC_50_ values reaching 0.082 and 0.023 μM, respectively [22] (Figure 2).

**Figure 3:**
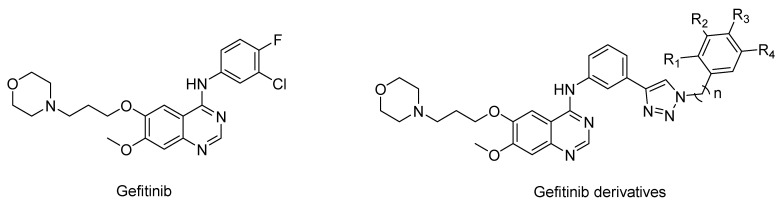
Structures of gefitinib and gefitinib derivatives.

To identify the compounds with potent inhibitory effects on EGFR wild-type lung cancer cells, we modified gefitinib through a click reaction by introducing a 1,2,3-triazole moiety to its structure (Figure 3). We aimed to evaluate its inhibitory effects on wild-type lung cancer cells. Using gefitinib as a positive control, we employed the cell counting kit-8 (CCK-8) method to assess the in vitro antitumor activity against EGFR wild-type lung cancer cells NCI-H1299, A549 and NCI-H1437. Additionally, we investigated the impact on tumor cell clone formation, migration capability and apoptosis.

## 2. Chemistry

**Scheme 1:**
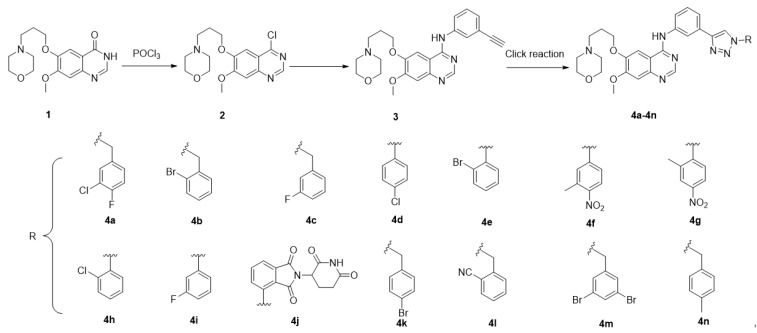
Synthesis route of gefitinib-1,2,3-triazole derivatives (compounds **4**).

The synthetic strategy for the preparation of the target molecules is illustrated through a literature review [23,24,25] in Scheme 1. The key intermediate 2 was obtained through chlorination with 7-methoxy-6-(3-morpholinopropoxy)quinazolin-4(3H)-one as the raw material, and subsequent condensation with 3-aminophenylethynyl to yield intermediate 3. Finally, 1,2,3-triazole gefitinib derivatives were synthesized through a copper(I)-catalyzed azide–alkyne cycloaddition reaction. The structures of the key intermediates and all target compounds were confirmed through nuclear magnetic resonance spectroscopy (^1^H NMR and ^13^C NMR) and high-resolution mass spectrometry (HRMS).

## 3. Results and Discussion

### 3.1. Compounds ***4a***–***4n*** Suppress Cancer Cell Viability

The cell inhibitory effects of compounds **4a**–**4n** on EGFR wild-type lung cancer cells NCI-H1299, NCI-H1437 and A549 were evaluated using the CCK-8 assay, with gefitinib as a positive control. The results showed that most compounds exhibited inhibitory effects on NCI-H1299, NCI-H1437 and A549 lung cancer cells, with compounds **4b** and **4c** showing the best performance. In the NCI-H1299 cell line, the IC_50_ values of **4b** and **4c** were 4.42 ± 0.24 μM and 4.60 ± 0.18 μM. In the NCI-H1437 cell line, the IC_50_ values of **4b** and **4c** were 1.56 ± 0.06 μM and 3.51 ± 0.05 μM. In the A549 cell line, the IC_50_ values of **4b** and **4c** were 3.94 ± 0.01 μM and 4.00 ± 0.08 μM. These values were better than the inhibitory effects of gefitinib on the three cell lines (IC_50_ values = 14.23 ± 0.08 μM, 20.44 ± 1.43 μM, and 15.11 ± 0.05 μM, respectively)(Table 1). In the L02 cell line, the IC_50_ values of **4b** and **4c** were 20.25 ± 1.26 μM and 29.38 ± 5.53 μM. In our study, we observed that the inhibition rate of **4b** and **4c** at a concentration of 4 μM on normal hepatic cells L02 is less than 20%. However, the inhibition rate on lung cancer cells was found to reach 50%, indicating a certain selective effect of these two compounds on tumor cells (Table 2). The results indicated that the structural modification of gefitinib was successful, and after the introduction of benzyl or phenyl groups, the compounds exhibited significantly different activities. When the triazole linkage was a benzyl group, the activity was better than that of the phenyl group. The presence of fluorine or bromine atoms in the ortho or meta position of the benzyl ring was favorable for enhancing the in vitro antitumor activity of the compounds.

### 3.2. ***4b*** and ***4c*** Induce Apoptosis in H1299 Cells

**Figure 4:**
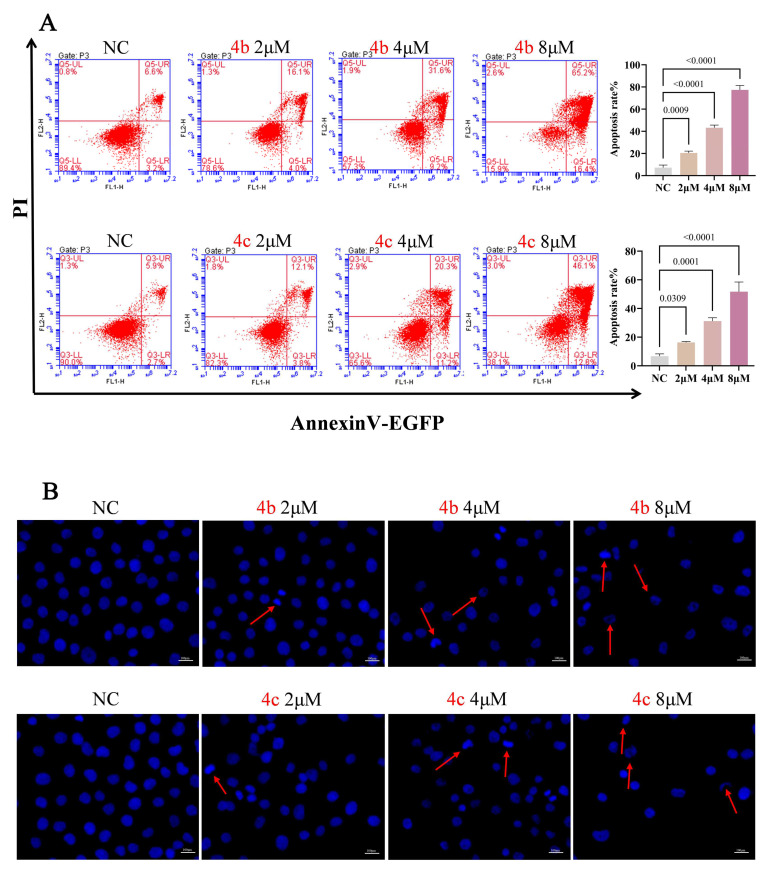
Cell apoptosis induced by **4b** and **4c** in H1299 cells. (**A**) Apoptosis quantification detected by Annexin V-EGFP/PI staining. (**B**) Cell apoptosis morphological changes detected by DAPI staining. Red arrows indicate DAPI-positive (apoptotic) cells. Images represent at least three independent experiments. Data were mean ± SD. *n* = 3 for each concentration. NC, normal control.

To elucidate whether the inhibitory effects of these compounds on cell proliferation and cytotoxicity are related to apoptosis, we conducted experiments with **4b** and **4c** which showed good inhibitory effects on the proliferation of the three cell lines. H1299 cells were treated with different concentrations of **4b** and **4c** for 48 h. The cells were stained with Annexin-V and propidium iodide (PI), and the proportion of apoptotic cells was detected by flow cytometry. As shown in Figure 4A, after treatment with compound **4b**, the total apoptotic cell proportions of H1299 were 20.43 ± 1.72% (2 μM), 43.2 ± 2.4% (4 μM) and 77.4 ± 3.97% (8 μM). After treatment with compound **4c**, the total apoptotic cell proportions of H1299 were 16.37 ± 0.57% (2 μM), 31.2 ± 2.36% (4 μM) and 51.77 ± 6.74% (8 μM). Compared with the normal control group, as the drug concentration increased the apoptotic proportion gradually increased (*p* < 0.01, *p* < 0.05). These results indicated that both **4b** and **4c** can significantly promote the apoptosis of H1299 cells in a concentration-dependent manner.

To further confirm the apoptosis-inducing activity of **4b** and **4c** in lung cancer cells, H1299 cells were treated with different concentrations of compounds **4b** and **4c** for 48 h, followed by 4′,6-diamidino-2-phenylindole (DAPI) staining. Fluorescence microscopy was used to observe the cells and their representative images are shown in Figure 4B. Compared with the normal control group, H1299 cells treated with compounds **4b** and **4c** exhibited typical apoptotic features. As the drug concentration increased, nuclear staining intensified, and even nuclear condensation and fragmentation was observed. Therefore, the apoptosis-related results suggest that both **4b** and **4c** can significantly promote apoptosis in human lung cancer cells (NCI-H1299) in a concentration-dependent manner.

### 3.3. ***4b*** and ***4c*** Suppress Metastasis of H1299 Cells

Malignant proliferation forms the basis for the occurrence and development of tumors. When tumor cells proliferate to a certain extent, they may detach from the primary site and metastasize to other organs in the body [26]. NSCLC is a highly metastatic cancer, and the low five-year survival rate is closely related to the high metastasis of lung cancer cells [27]. Therefore, the metastasis of tumor cells is an important cause of death in cancer patients.

**Figure 5:**
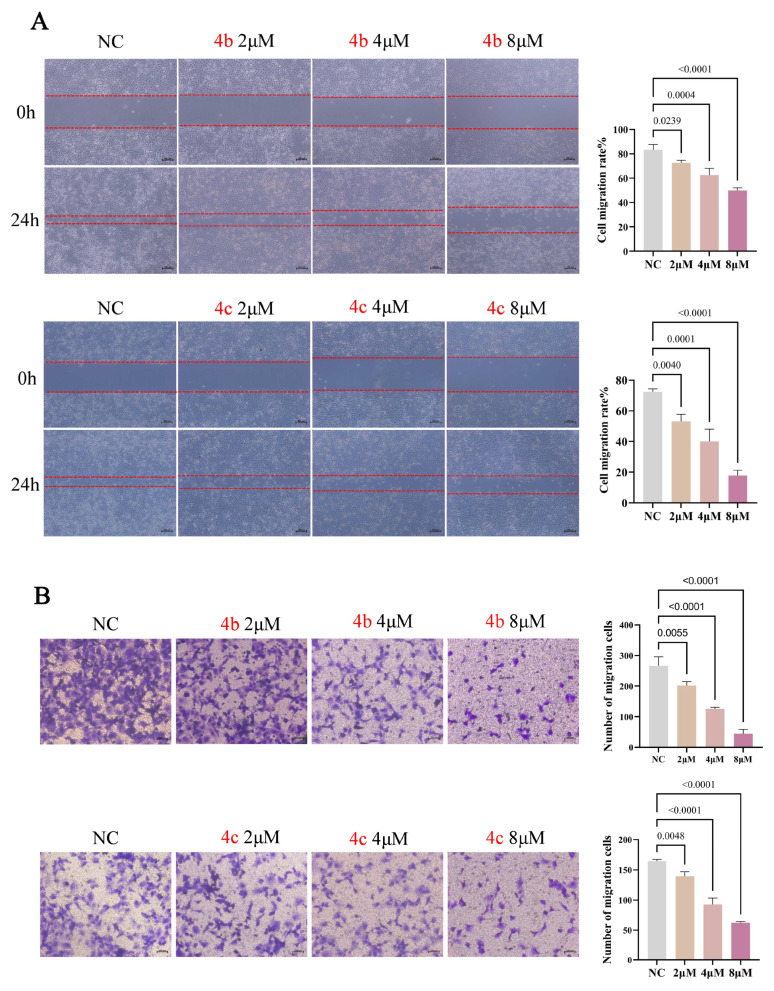
Cell migration inhibition induced by **4b** and **4c** in H1299 cells. (**A**) In vitro cell scratch assays. Photographs of cells at 0 and 24 h after treatment with different concentrations of drugs (scale bar = 100 μm). (**B**) Transwell migration assays. Migration of cells to the lower surface of the chamber was determined by crystal violet staining. Data were mean ± SD. *n* = 3 for each concentration. NC, normal control.

To investigate the inhibitory effects of compounds **4b** and **4c** on the migration of H1299 cells, cell scratch and transwell assays were employed. Figure 5A shows the results of the cell scratch assay, indicating that after treatment with compound **4b**, the migration closure rates of H1299 cells were 72.59 ± 2.12% (2 μM), 62.42 ± 5.50% (4 μM) and 49.79 ± 2.22% (8 μM). After treatment with compound **4c**, the migration closure rates of H1299 cells were 53.26 ± 4.51% (2 μM), 40.04 ± 8.02% (4 μM) and 17.90 ± 3.46% (8 μM). The cell scratch closure rates in the compound-treated groups were significantly lower than those in the normal controlgroup and exhibited concentration dependence (*p* < 0.01, *p* < 0.05). Transwell migration experiment results, as shown in Figure 5B, indicated a significant reduction in the number of cells crossing the microporous membrane to reach the lower surface of the chamber in the compound-treated groups compared to the normal control group (*p* < 0.01). In summary, compounds **4b** and **4c** can inhibit the migration ability of H1299 cells in a concentration-dependent manner.

### 3.4. ***4b*** and ***4c*** Inhibit the Clonogenic Ability of H1299 Cells

**Figure 6:**
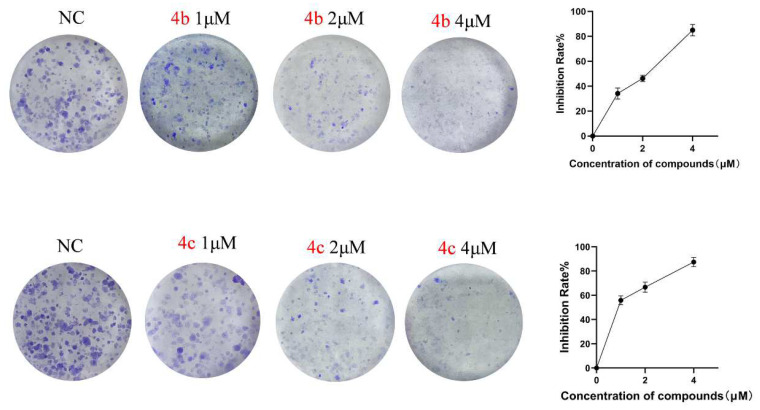
Colony formation pictures of the H1299 treated with **4b** and **4c**. NC, normal control.

The colony formation assay was employed to assess the ability of adherent cells to proliferate and form cell colonies on a flat surface [28]. It is an important method for detecting cell proliferation ability. In our study, the colony formation assay was used to further confirm that compounds **4b** and **4c** can inhibit the proliferation of H1299 cells (as shown in Figure 6). Cells were treated with low, medium and high concentrations (1, 2, 4 μM) of **4b** and **4c** for seven days. By counting the number of cell colonies, the analysis of the proliferation inhibition rate showed a significant reduction in the number of cell colonies in the compound-treated groups, and this effect was concentration-dependent. Even at the lowest concentration of 1 μM, compounds started to significantly inhibit the formation of H1299 cell colonies. Particularly at a concentration of 4 μM, the formation of colonies was almost completely inhibited, indicating that compounds **4b** and **4c** exert strong anti-proliferative effects on H1299 cells.

### 3.5. ***4b*** and ***4c*** Trigger Apoptosis via the Apoptosis Signaling Pathway

We conducted an in-depth investigation into the mechanism by which compounds **4b** and **4c** induce apoptosis in cells. The B-cell lymphoma 2 (Bcl-2) protein family is located upstream in the apoptosis signaling pathway. Bcl-2 proteins play an anti-apoptotic role by inhibiting the release of cytochrome C (cyt-c) from the mitochondria. Many anticancer drugs induce apoptosis by downregulating Bcl-2 [29]. When cell apoptosis occurs, the expression level of Bcl-2 is inhibited, cyt-c is released and, along with the apoptosis protease-activating factor, forms an apoptosome, initiating a cascade reaction of the caspase protein family. Initially, the apoptosome promotes the activation of caspase 9, which in turn activates caspase 3 forming the caspase 3 cleavage body. The caspase 3 cleavage body further activates the downstream PARP protein, ultimately promoting cell apoptosis [30].

**Figure 7:**
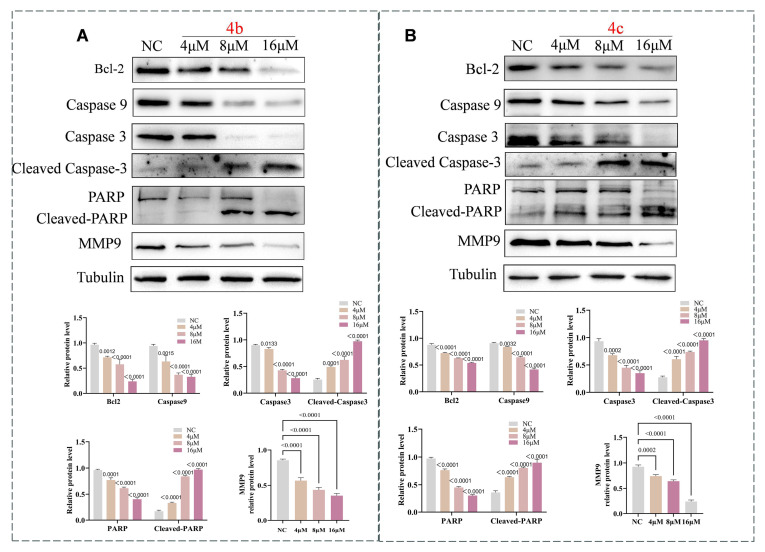
Assessment of apoptosis and migration induced by compounds **4b** and **4c**. (**A**) Western blotting results of the protein levels in H1299 cells treated with 4, 8 or 16 μM of **4b** compounds. Numbers above each lane indicated the relative expression level of the protein. (**B**) Western blotting results of the protein levels in H1299 cells treated with 4, 8 or 16 μM of **4c** compounds. Numbers above each lane indicated the relative expression level of the protein. NC, normal control.

Therefore, after treating H1299 cells with different concentrations of compounds for 48 h, cell proteins were extracted and Western blot analysis revealed a concentration-dependent decrease in the expression levels of Bcl-2, caspase 9, caspase 3 and PARP proteins in the groups treated with compounds **4b** and **4c** (Figure 7). Simultaneously, the expression levels of cleaved caspase 3 and cleaved PARP proteins increased. These results suggest that compounds **4b** and **4c** may effectively induce apoptosis in NSCLC cells through the Bcl-2/caspase3/PARP signaling pathway, and the apoptotic effect in the high drug concentration group was significantly stronger than that in the low drug concentration and normal control groups (*p* < 0.01, *p* < 0.05).

There is existing research indicating that matrix metalloproteinase 9 (MMP9) plays a crucial role in angiogenesis and cell migration [31]. In NSCLC tissues, the protein expression level of MMP9 is significantly higher than that in normal adjacent tissues. This suggests a close correlation between the high expression of MMP9 and malignant metastasis of lung cancer, as well as a poor prognosis [32]. To further investigate whether compounds regulate the migratory ability of NSCLC by modulating MMP9 protein levels, Western blot analysis showed a decrease in MMP9 protein expression with increasing drug concentration. Therefore, compounds **4b** and **4c** may inhibit the migratory ability of NSCLC cells by downregulating MMP9 protein expression.

## 4. Conclusions

This study was based on the structure of gefitinib. Fourteen structurally novel gefitinib derivatives were obtained through click reactions. Simultaneously, the synthesized 1,2,3-triazole derivatives were evaluated for their antitumor activity against wild-type lung cancer cells. The synthetic method was simple and rapid, and the compounds were characterized and confirmed structurally. The in vitro antitumor activity of the fourteen compounds was evaluated using three cancer cell lines. The results showed that compounds **4b** and **4c** exhibited strong antiproliferative activity in three NSCLC cell lines. Through cellular function and mechanism studies, it was found that compounds **4b** and **4c** downregulated the protein expression of Bcl-2, caspase 9 and MMP9, upregulated the protein expression of cleaved caspase 3 and cleaved PARP, inhibited tumor cell proliferation and migration and promoted cell apoptosis. This research lays the foundation for future in vivo experiments and clinical applications of these novel gefitinib derivatives.

## 5. Experimental

### 5.1. Materials and Chemistry

All reagents and solvents obtained from commercially available source were used without further treatment. ^1^H NMR and ^13^C NMR spectra were acquired in DMSO-*d*_6_ or Methanol-*d*_4_ solution with a Bruker 600 spectrometer (Bruker, Billerica, MA, USA). High-resolution mass spectra (HRMS) measurements were carried out using a Bruker Compact mass spectrometer.

Lung cell lines A549, NCI-H1437 and NCI-H1299 were purchased from Procell Life Science and Technology Co., Ltd. (Wuhan, China). Ham’s F-12K medium and fetal bovine serum (FBS) was purchased from Procell Life Science and Technology Co., Ltd. (Wuhan, China). Human normal hepatocyte cell line L02 (College of Basic Medicine and Forensic Medicine, Henan University of Science and Technology, Luoyang, China) was cultured in RPMI-1640 medium. RPMI-1640 medium and 4′,6-diamidino-2-phenylindole (DAPI) were purchased from Solarbio Science Technology (Bejing, China). Cell counting kit-8 and Annexin V-EGFP apoptosis detection kit were obtained from Beyotime Biotechnology (Shanghai, China). The primary antibodies against Caspase3 (1:500), PARP/cleaved-PARP (1:750) and MMP9 (1:1000) were obtained from Wan lei Biotechnology (Shenyang, China). The primary antibodies against Alpha Tubulin (1:2000), cleaved-Caspase3 (1:500) and Caspase9 (1:300) was purchased from Proteintech (Wuhan, China). Bcl-2 (1:1000), HRP-conjugated affinipure goat anti-rabbit IgG (1:1000) was purchased from Cell Signaling Technology (Danvers, MA, USA).

#### 5.1.1. Preparation of 4-(3-((4-chloro-7-methoxyquinazolin-6yl)oxy)propyl)morpholine

A mixture of Compound **1** (3.0 g, 9.0 mmol) in POCI_3_ (10.0 mL) was allowed to stir at 120 °C for 12 h. Progress of reaction was monitored by TLC. After completion, the reaction mixture was cooled to RT, diluted with cold water (300 mL) and allowed to stir for 10 min. The reaction mixture was adjusted to neutral pH with saturated sodium bicarbonate solution and extracted with dichloromethane. It was then dried with anhydrous magnesium sulfate, concentrated to afford **2** (2.0 g, 65%) as a yellow solid. Mp: 116–119 °C. ^1^H NMR (400 MHz, DMSO-*d*_6_) *δ* 8.86 (s, 1H), 7.43 (s, 1H), 7.38 (s,1H), 4.23 (t, 2H), 3.95 (s, 3H), 3.57 (t, 4H), 2.43 (t, 2H), 2.37 (s, 4H), 2.03–1.92 (m, 2H).

#### 5.1.2. Preparation of *N*-(3-ethynylphenyl)-7-methoxy-6-(3-morpholinopropoxy)quinazolin-4-amine

A suspension of Compound **2** (1.5 g, 4.5 mmol) and *m*-acetylenyl aniline (1.56 g, 13.4 mmol) in isopropanol (40 mL) was allowed to stir at 100 °C for 6 h. Reaction mixture was cooled to RT, diluted with cold water (50 mL) and allowed to stir at RT for 10 min. Solid was filtered, washed with water and dried under vacuum to afford solid product 3 (1.19 g, 64%). ^1^HNMR (400 MHz, DMSO-*d*_6_) *δ* 11.22 (s, 1H), 8.78 (s, 1H), 8.50 (s, 1H), 7.99 (s, 1H), 7.91 (d, *J* = 8.2 Hz, 1H), 7.48 (t, *J* = 7.9 Hz, 1H), 7.37 (d, *J* = 8.8 Hz, 2H), 4.40 (s, 2H), 4.28 (s, 1H), 3.96–3.79 (m, 7H), 3.39–3.160 (m, 6H), 2.35 (s, 2H).

#### 5.1.3. General Procedure for the Preparation of Compound **4**

Aryl-azido (150 mg, 0.7 mmol) and **3** (250 mg, 0.56 mmol) were added to 15 mL mixed solvent (water/tert-butanol/THF = 1:1:1). Copper sulfate pentahydrate (14 mg, 0.1 mmol) and sodium ascorbate (23 mg, 0.1 mmol) were added, and the mixture was stirred at 85 °C for 12 h. After the completion of the reaction (monitored by TLC), the mixture was extracted with dichloromethane (15 mL × 3). The combined organic phase was washed successively with brine, dried over sodium sulfate and concentrated in vacuo. The residue was purified through column chromatography (CH_2_Cl_2_/MeOH = 20:1) to give the desired compound **4** as a crystalline powder. The nuclear magnetic resonance spectroscopy (^1^H NMR and ^13^C NMR) and high-resolution mass spectrometry (HRMS) of compounds **4a**–**4n** are Supplementary Materials provided as data.

### 5.2. Synthesis of Snalogues ***4a***–***4n***

*N-(3-(1-(3-Chloro-4-fluorobenzyl)-1H-1,2,3-triazol-4-yl)phenyl)-7-methoxy-6-(3-morpholinopropoxy)quinazolin-4-amine* (**4a**): Brown solid, yield: 85%, Purity 96.1%; m.p. 192–193 °C, ^1^H NMR (400 MHz, DMSO-*d*_6_) *δ* 9.60 (s, 1H), 8.69 (s, 1H), 8.49 (s, 1H), 8.27 (t, *J* = 1.9 Hz, 1H), 7.97–7.87 (m, 2H), 7.69 (dd, *J* = 7.1, 2.1 Hz, 1H), 7.59–7.50 (m, 1H), 7.49–7.39 (m, 3H), 7.21 (s, 1H), 5.68 (s, 2H), 4.21 (t, *J* = 6.4 Hz, 2H), 3.95 (s, 3H), 3.59 (t, *J* = 4.6 Hz, 4H), 2.49–2.47 (m, 2H), 2.40–2.39 (m, 4H), 2.10–1.88 (m, 2H). ^13^C NMR (100 MHz, DMSO) *δ* 158.7, 156.8, 156.3, 154.8, 153.3, 148.7, 147.2, 140.5, 134.3, 134.2 (d, *J* = 3.7 Hz), 131.2, 131.0, 129.6, 122.4, 122.2, 120.8, 120.3 120.1, 119.3, 117.9, 117.7, 107.7, 103.1, 67.6, 66.6, 56.3, 55.5, 53.9, 52.2, 31.8, 26.3. HR MS (ESI) *m*/*z*: calcd for C_31_H_31_ClFN_7_O_3_ [M + H]^+^ 604.2234, found 604.2234.

*N-(3-(1-(2-Bromobenzyl)-1H-1,2,3-triazol-4-yl)phenyl)-7-methoxy-6-(3-morpholinopropoxy)quinazolin-4-amine* (**4b**): Yellow solid, yield 84%, Purity 95.1%; m.p. 165–166 °C, ^1^H NMR (400 MHz, DMSO- *d*_6_) *δ* 9.65 (s, 1H), 8.63 (s, 1H), 8.49 (s, 1H), 8.29 (s, 1H), 8.06–7.83 (m, 2H), 7.72 (d, *J* = 8.0 Hz, 1H), 7.62–7.54 (m, 1H), 7.51–7.39 (m, 2H), 7.39–7.29 (m, 1H), 7.28–7.17 (m, 2H), 5.76 (s, 2H), 4.25 (t, 2H), 3.95 (s, 3H), 3.75–3.62 (m, 4H), 2.97–2.66 (m, 4H), 2.26–2.03 (m, 2H). ^13^C NMR (100 MHz, DMSO) *δ* 156.9, 154.8, 153.3, 148.5, 147.3, 146.9, 140.5, 135.3, 133.4, 131.3, 131.0, 130.9, 129.5, 128.8, 123.4, 122.5, 122.4, 120.9, 119.4, 109.4, 107.8, 103.5, 67.4, 65.5, 56.3, 55.0, 53.6, 53.0, 25.1. HR MS (ESI) *m*/*z*: calcd for C_31_H_32_BrN_7_O_3_ [M + H]^+^ 630.1823, found 630.1824.

*N-(3-(1-(3-Fluorobenzyl)-1H-1,2,3-triazol-4-yl)phenyl)-7-methoxy-6-(3-morpholinopropoxy)quinazolin-4-amine* (**4c**): White solid, yield 75%, Purity 97.0%; m.p. 202–203 °C, ^1^H NMR (400 MHz, DMSO- *d*_6_) *δ* 9.64 (s, 1H), 8.74 (s, 1H), 8.53 (s, 1H), 8.32 (s, 1H), 7.95 (d, *J* = 10.0 Hz, 2H), 7.61 (d, *J* = 7.7 Hz, 1H), 7.54–7.44 (m, 2H), 7.30–7.19 (m, 4H), 5.74 (s, 2H), 4.24 (t, *J* = 6.3 Hz, 2H), 3.98 (s, 3H), 3.63–3.59 (m, 4H), 2.48–2.40 (m, 4H), 2.10–1.98 (m, 2H). ^13^C NMR (100 MHz, DMSO) *δ* 163.9, 161.4, 156.8, 154.8, 153.3, 148.7, 147.4, 147.2, 140.6, 139.1 (d, *J* = 7.4 Hz), 131.4, 129.5, 124.5 (d, *J* = 2.8 Hz), 124.4, 122.4, 122.3 (d, *J* = 15.3 Hz), 120.8, 119.3, 115.6, 115.5, 115.2, 109.4, 107.7, 103.1, 67.6, 66.6, 56.3, 55.4, 53.9, 52.8, 26.3. HR MS (ESI) *m*/*z*: calcd for C_31_H_32_FN_7_O_3_ [M + H]^+^ 570.2623, found 570.2625.

*N-(3-(1-(4-Chlorophenyl)-1H-1,2,3-triazol-4-yl)phenyl)-7-methoxy-6-(3-morpholinopropoxy)quinazolin-4-amine* (**4d**): White solid, yield 60%, Purity 96.9%; m.p. 162–163 °C, ^1^H NMR (400 MHz, Methanol-*d*_4_) *δ* 8.57 (s, 1H), 8.38 (s, 1H), 8.12 (t, *J* = 1.9 Hz, 1H), 7.79–7.67 (m, 3H), 7.64 (s, 1H), 7.55 (d, *J* = 7.7 Hz, 1H), 7.50–7.44 (m, 2H), 7.39 (t, *J* = 7.9 Hz, 1H), 7.06 (s, 1H), 4.18 (t, *J* = 6.3 Hz, 2H), 3.92 (s, 3H), 3.65 (t, *J* = 4.6 Hz, 4H), 2.54 (t, *J* = 7.5 Hz, 2H), 2.49–2.42 (m, 4H), 2.10–1.96 (m, 2H). ^13^C NMR (100 MHz, MeOD) *δ* 157.3, 155.3, 152.8, 149.0, 148.3, 146.5, 139.7, 135.5, 134.6, 130.4, 129.8, 129.4, 122.9, 121.6, 121.5, 119.8, 118.8, 106.2, 102.5, 67.4, 66.5, 55.7, 55.5, 53.5, 25.9. HR MS (ESI) *m*/*z*: calcd for C_30_H_30_ClN_7_O_3_ [M + H]^+^ 572.2171, found 572.2175.

*N-(3-(1-(2-Bromophenyl)-1H-1,2,3-triazol-4-yl)phenyl)-7-methoxy-6-(3-morpholinopropoxy)quinazolin-4-amine* (**4e**): Yellow solid, yield 56%, Purity 97.1%; m.p. 175–176 °C, ^1^H NMR (400 MHz, DMSO-*d*_6_) *δ* 9.68 (s, 1H), 9.03 (s, 1H), 8.38 (s, 1H), 8.04–7.73 (m, 4H), 7.72–7.42 (m, 4H), 7.57–7.42 (m, 1H) 4.32–4.21 (m, 2H), 4.10–3.95 (m, 3H), 3.62–3.52 (m, 5H), 2.45–2.35 (m, 4H), 2.01 (t, *J* = 6.7 Hz, 2H). ^13^C NMR (100 MHz, DMSO) *δ* 149.2, 146.9, 140.6, 137.1, 136.7, 135.0, 134.1, 132.6, 131.0, 130.4, 129.7, 129.5, 129.2, 124.2, 122.8, 121.1, 120.5, 120.2, 119.6, 119.5, 67.6, 66.6 56.5, 55.4, 53.9, 34.7, 26.8, 26.3. HR MS (ESI) *m*/*z*: calcd for C_30_H_30_BrN_7_O_3_ [M + H]^+^ 616.1666, found 616.1667.

*7-Methoxy-N-(3-(1-(3-methyl-4-nitrophenyl)-1H-1,2,3-triazol-4-yl)phenyl)-6-(3-morpholinopropoxy)quinazolin-4-amine* (**4f**): Yellow solid, yield 55%, Purity 97.1%; m.p. 175–176 °C, ^1^H NMR (400 MHz, DMSO- *d*_6_) *δ* 9.66 (s, 1H), 9.09 (s, 1H), 8.53 (s, 1H), 8.42 (t, *J* = 1.9 Hz, 1H), 8.27–8.15 (m, 1H), 8.00–7.84 (m, 3H), 7.79–7.70 (m, 1H), 7.67–7.64 (m, 1H), 7.53 (t, *J* = 7.9 Hz, 1H), 7.22 (s, 1H), 4.22 (t, *J* = 6.3 Hz, 2H), 3.95 (s, 3H), 3.59 (t, *J* = 4.6 Hz, 4H), 2.53–2.48 (m, 2H), 2.45–2.39 (m, 4H), 2.27 (s, 3H), 2.06–1.99 (m, 2H). ^13^C NMR (100 MHz, DMSO) *δ* 156.8, 154.8, 153.2, 151.2, 148.7, 147.3, 140.7, 138.2, 131.5, 130.8, 129.6, 128.8, 128.5, 126.2, 124.3, 122.8, 121.0, 119.6, 107.8, 103.1, 67.6, 66.6, 60.2, 56.3, 55.4, 53.9, 26.3, 21.2, 14.5. HR MS (ESI) *m*/*z*: calcd for C_31_H_32_N_8_O_5_ [M + H]^+^ 597.2568, found 597.2569.

*7-Methoxy-N-(3-(1-(2-methyl-3-nitrophenyl)-1H-1,2,3-triazol-4-yl)phenyl)-6-(3-morpholinopropoxy)quinazolin-4-amine* (**4g**): Brown solid, yield 62%, Purity 96.9%; m.p. 177–178 °C, ^1^H NMR (400 MHz, DMSO-*d*_6_) *δ* 9.63 (s, 1H), 9.06 (s, 1H), 8.50 (s, 1H), 8.43–8.37 (m, 1H), 8.22–8.16 (m, 1H), 7.98–7.88 (m, 3H), 7.80–7.69 (m, 1H), 7.68–7.62 (m, 1H), 7.51 (t, *J* = 7.9 Hz, 1H), 7.21 (s, 1H), 4.22 (t, *J* = 6.4 Hz, 2H), 3.95 (s, 3H), 3.59 (t, *J* = 4.6 Hz, 4H), 2.45–2.38 (m, 4H),2.26 (m, 3H), 2.08–1.96 (m, 2H). ^13^C NMR (100 MHz, DMSO) *δ* 154.8, 148.8, 147.0, 140.6, 135.0, 132.3, 131.1, 130.9, 129.6, 129.1, 129.0, 128.9, 124.2, 122.8, 121.1, 119.6, 103.3, 67.6, 66.6, 56.3, 55.4, 53.9, 34.7, 32.0, 28.9, 26.8, 26.3, 22.9, 14.4. HR MS (ESI) *m*/*z*: calcd for C_31_H_32_N_8_O_5_ [M + H]^+^ 597.2568, found 597.2568.

*N-(3-(1-(2-Chlorophenyl)-1H-1,2,3-triazol-4-yl)phenyl)-7-methoxy-6-(3-morpholinopropoxy)quinazolin-4-amine* (**4h**): Brown solid, yield 65%, Purity 96.9%; m.p. 167–168 °C, ^1^H NMR (400 MHz, DMSO- *d*_6_) *δ* 9.67 (s, 1H), 9.49 (s, 1H), 8.41–8.37 (m, 1H), 8.31–8.26 (m, 1H), 8.24–8.18 (m, 1H), 8.11–8.06 (m, 1H), 7.99–7.90 (m, 2H), 7.69–7.62 (m, 1H), 7.54 (t, *J* = 7.9 Hz, 1H), 7.23 (s, 1H), 4.21 (t, *J* = 6.4 Hz, 2H), 3.96 (s, 3H), 3.60 (t, *J* = 4.6 Hz, 4H), 2.66 (s, 3H), 2.42 (s, 4H), 2.10–1.95 (m, 2H). ^13^C NMR (100 MHz, DMSO) *δ* 156.8, 154.8, 148.8, 148.3, 148.2, 140.7, 139.7, 136.1, 130.5, 129.7, 127.2, 123.6, 123.0, 121.0, 120.4, 119.5, 118.5, 103.1, 67.6, 66.6, 56.3, 55.5, 53.9, 29.5, 26.3, 22.6, 20.4, 14.4. HR MS (ESI) *m*/*z*: calcd for C_30_H_30_ClN_7_O_3_ [M + H]^+^ 572.2171, found 572.2170.

*N-(3-(1-(3-Fluorophenyl)-1H-1,2,3-triazol-4-yl)phenyl)-7-methoxy-6-(3-morpholinopropoxy)quinazolin-4-amine* (**4i**): White solid, yield 66%, Purity 97.9%; m.p. 176–178 °C, ^1^H NMR (400 MHz, DMSO- *d*_6_) *δ* 9.64 (s, 1H), 9.38 (s, 1H), 8.55 (s, 1H), 8.43–8.33 (m, 1H), 7.98–7.84 (m, 4H), 7.76–7.62 (m, 2H), 7.53 (t, *J* = 7.9 Hz, 1H), 7.42–7.31 (m, 1H), 7.23 (s, 1H), 4.23 (t, *J* = 6.4 Hz, 2H), 3.96 (s, 3H), 3.60 (t, *J* = 4.6 Hz, 4H), 2.56–2.52 (m, 2H), 2.48–2.37 (m, 4H), 2.07–1.96 (m, 2H). ^13^C NMR (100 MHz, DMSO) *δ* 164.2, 161.7, 156.9, 154.9, 148.8, 148.0, 140.7, 138.3 (d, *J* = 10.8 Hz), 132.4 (d, *J* = 9.4 Hz), 130.8, 129.6,122.9, 121.0, 120.3, 119.6, 116.4, 116.3, 116.0, 115.8, 108.1, 107.8, 103.3, 67.7, 66.6, 56.3, 55.4, 53.9, 26.3. HR MS (ESI) *m*/*z*: calcd for C_30_H_30_BrN_7_O_3_ [M + H]^+^ 570.2628, found 570.2629.

*2-(2,6-Dioxopiperidin-3-yl)-4-(4-(3-((7-methoxy-6-(3-morpholinopropoxy)quinazolin-4-yl)amino)phenyl)-1H-1,2,3-triazol-1-yl)isoindoline-1,3-dione* (**4j**): White solid, yield 34%, Purity 96.9%; m.p. 187–188 °C, ^1^H NMR (400 MHz, DMSO-*d*_6_) *δ* 11.16 (s, 1H), 9.67 (s, 1H), 9.20 (s, 1H), 8.35 (d, *J* = 1.9 Hz, 1H), 8.28–8.24 (m, 1H), 8.15–8.13 (m, 2H), 8.01–7.88 (m, 2H), 7.64 (d, *J* = 7.6 Hz, 1H), 7.53 (t, *J* = 7.9 Hz, 1H), 5.26–5.10 (m, 1H), 4.22 (t, *J* = 6.3 Hz, 2H), 3.97 (s, 3H), 3.59 (t, *J* = 4.6 Hz, 4H), 2.96–2.79 (m, 1H), 2.67–2.52 (m, 3H), 2.46–2.34 (m, 4H), 2.10–1.93 (m, 3H). ^13^C NMR (100 MHz, DMSO) *δ* 173.2, 170.0, 166.4, 165.1, 163.3, 158.3,156.6, 156.5, 155.0, 148.8, 146.8, 140.7, 137.0, 133.2, 131.5, 130.7, 129.7, 124.8, 124.4, 123.2, 122.9, 121.0, 119.5, 103.5, 81.1, 67.6, 66.6, 56.4, 55.4, 53.8, 49.7, 36.2, 31.3, 26.3, 22.3. HR MS (ESI) *m*/*z*: calcd for C_37_H_35_N_9_O_7_ [M + H]^+^ 718.2732, found 728.2731.

*N-(3-(1-(4-Bromobenzyl)-1H-1,2,3-triazol-4-yl)phenyl)-7-methoxy-6-(3-morpholinopropoxy)quinazolin-4-amine* (**4k**): Yellow solid, yield 71%, Purity 97.7%; m.p. 177–178 °C, ^1^H NMR (400 MHz, DMSO- *d*_6_) *δ* 9.59 (s, 1H), 8.65 (s, 1H), 8.25 (d, *J* = 1.9 Hz, 1H), 7.97–7.85 (m, 2H), 7.65–7.58 (m, 2H), 7.57–7.52 (m, 1H), 7.48–7.42 (m, 1H), 7.37–7.29 (m, 2H), 5.66 (s, 2H), 4.21 (t, *J* = 6.3 Hz, 2H), 3.95 (s, 3H), 3.59 (t, *J* = 4.6 Hz, 5H), 2.45–2.37 (m, 4H), 2.07–1.95 (m, 3H). ^13^C NMR (100 MHz, DMSO) *δ* 156.7, 154.8, 148.8, 147.2, 140.5, 135.9, 132.2, 131.3, 130.7, 129.5, 122.4, 122.2, 121.9, 120.8, 119.3, 103.3, 67.6, 66.6, 56.3, 55.4, 53.8, 52.8, 31.7, 29.5, 29.0, 27.0, 26.2. HR MS (ESI) *m*/*z*: calcd for C_31_H_32_BrN_7_O_3_ [M + H]^+^ 630.1823, found 630.1819.

*2-((4-(3-((7-Methoxy-6-(3-morpholinopropoxy)quinazolin-4-yl)amino)phenyl)-1H-1,2,3-triazol-1-yl)methyl)benzonitrile* (**4l**): Yellow solid, yield 67%, Purity 95.7%; m.p. 167–168 °C, ^1^H NMR (400 MHz, DMSO- *d*_6_) *δ* 9.60 (s, 1H), 8.69 (s, 1H), 8.50 (s, 1H), 8.27 (t, *J* = 1.9 Hz, 1H), 8.00–7.87 (m, 3H), 7.84–7.72 (m, 1H), 7.66–7.37 (m, 4H), 7.22 (s, 1H), 5.89 (s, 2H), 4.21 (t, *J* = 6.3 Hz, 2H), 3.95 (s, 3H), 3.68–3.56 (m, 4H), 2.48–2.37 (m, 4H), 2.14–1.88 (m, 2H). ^13^C NMR (100 MHz, DMSO) *δ* 156.8, 154.8, 150.0, 148.7, 147.1, 140.6, 139.2, 134.4, 133.9, 131.2, 129.7, 129.6, 129.5, 122.6, 122.5, 120.8, 119.4, 117.5, 116.0, 111.7, 103.2, 71.0, 67.6, 66.6, 56.3, 55.4, 55.3, 53.8, 51.7, 26.2. HR MS (ESI) *m*/*z*: calcd for C_32_H_32_N_8_O_3_ [M + H]^+^ 577.2670, found 577.2671.

*N-(3-(1-(3,5-Dibromobenzyl)-1H-1,2,3-triazol-4-yl)phenyl)-7-methoxy-6-(3-morpholinopropoxy)quinazolin-4-amine* (**4m**): Yellow solid, yield 75%, Purity 96.6%; m.p. 179–180 °C, ^1^H NMR (400 MHz, DMSO- *d*_6_) *δ* 9.61 (s, 1H), 8.71 (s, 1H), 8.27 (s, 1H), 8.06 (s, 1H), 7.94–7.87 (m, 2H), 7.86 (s, 1H), 7.70–7.61 (m, 2H), 7.57 (d, *J* = 7.6 Hz, 1H), 7.46 (t, *J* = 7.8 Hz, 1H), 5.69 (s, 2H), 4.21 (t, *J* = 4.6 Hz, 2H), 4.00–3.87 (m, 3H), 3.59 (t, *J* = 4.6 Hz, 4H), 2.47–2.33 (m, 4H), 2.07–1.94 (m, 2H). ^13^C NMR (100 MHz, DMSO) *δ* 154.7, 148.8, 147.2, 147.0, 140.8, 140.6, 133.7, 131.2, 130.7, 129.5, 123.2, 122.5, 122.4, 120.8, 119.4, 104.6, 103.5, 92.2, 67.6, 66.6, 56.4, 55.4, 53.9, 52.0, 26.3. HR MS (ESI) *m*/*z*: calcd for C_31_H_31_Br_2_N_7_O_3_ [M + H]^+^ 710.0907, found 710.0906.

*7-methoxy-N-(3-(1-(4-methylbenzyl)-1H-1,2,3-triazol-4-yl)phenyl)-6-(3-morpholinopropoxy)quinazolin-4-amine* (**4n**): Yellow solid, yield 62%, Purity 96.5%; m.p. 168–169 °C, ^1^H NMR (400 MHz, DMSO-*d*_6_) *δ* 9.60 (s, 1H), 8.62 (s, 1H), 8.25 (s, 1H), 8.13–8.04 (m, 1H), 7.90 (d, *J* = 7.2 Hz, 1H), 7.74–7.65 (m, 1H), 7.55 (d, *J* = 7.6 Hz, 1H), 7.44 (t, *J* = 7.8 Hz, 1H), 7.35–7.14 (m, 5H), 5.60 (s, 2H), 4.25–4.15 (m, 3H), 3.98 (s, 3H), 3.66–3.51 (m, 4H), 2.46–2.39 (m, 4H), 2.32–2.30 (m, 1H), 2.29 (s, 3H), 2.10–1.96 (m, 2H). ^13^C NMR (100 MHz, DMSO) *δ* 148.9, 147.1, 140.5, 138.0, 133.5, 131.4, 129.8, 129.7, 129.5, 129.1, 129.0, 128.5, 128.4, 122.4, 122.0, 120.8, 119.3, 67.6, 66.6, 65.5, 56.4, 55.4, 53.8, 53.3, 30.5, 26.3, 21.2, 19.1, 14.0. HR MS (ESI) *m*/*z*: calcd for C_32_H_35_N_7_O_3_ [M + H]^+^ 566.2874, found 566.2873.

### 5.3. Biological Study

#### 5.3.1. Cell Culture

NCI-H1299, NCI-H1437 and L02 cells were cultured in a humidified incubator (Thermo Scientific^™^ Forma^™^ Direct Heat CO_2_ Incubator, Waltham, MA, USA) at 37 °C with 5% CO_2_ in RPMI-1640 supplemented with 10% fetal bovine serum (FBS) and 1% Penicillin Streptomycin. A549 cells were cultured in a humidified incubator at 37 °C with 5% CO_2_ in Ham’s F-12K supplemented with 10% FBS and 1% Penicillin Streptomycin.

#### 5.3.2. CCK-8 Assay to Assess Cell Proliferation and Cytotoxicity

Three types of lung cells and L02 cells were seeded into 96-well plate at a density of 5 × 10^3^ cells per well in the logarithmic growth phase and cultured at 37 °C. The cells were then treated initially with different concentrations of compounds **4a**–**4n** (0, 2, 4, 8, 16 and 32 μM) for an additional 48 h, with three replicate wells each. Next, cell viability was determined according to the instructions of the CCK-8 assay. Then, 10 μL CCK-8 solution was added to each well of the plate and incubated for 1–4 h in the incubator. The absorbance was measured at 450 nm using a microplate reader (Bio-Tek, Winooski, VT, USA). The percentage of viable cells was measured using the following formula where three independent experiments were performed: [(A450_sample_ − A450_blank_)/(A450_control_ − A450_blank_)] × 100%.

#### 5.3.3. Flow Cytometry Detection for Cell Apoptosis

H1299 cells were seeded in a 6-well plate at a density of 1 × 10^5^ cells/well and incubated for 24 h. The following day, the medium was replaced with fresh medium containing **4b** or **4c** (2, 4 and 8 μM) and cells were incubated for an additional 48 h. Cell apoptosis was detected using Annexin V-EGFP/PI apoptosis detection kit (Beyotime Biotechnology, Shanghai, China) by flow cytometry. Subsequently, 5 μL of EGFR Annexin V and 10 μL of PI was added to the cell suspension, gently vortexed and incubated at room temperature in the dark for 20 min. Then, single-cell suspension was prepared followed by flow cytometry (BD Accuri™ C6 Plus, BD Biosciences, Franklin Lake, NJ, USA).

#### 5.3.4. DAPI Staining for Cell Apoptosis

H1299 (1.5 × 10^4^–2 × 10^4^/well) cells were seeded in 24-well plates for 24 h and then treated with **4b** or **4c** at the concentrations of 2, 4 and 8 μM for 48 h. The cells were fixed for 30 min at room temperature in 4% paraformaldehyde and washed three times in PBS. Finally, every well was stained with DAPI (10 μg/mL) at room temperature for 30 min and washed at least three times in PBS. The fluorescence microscope was used to observe the morphological changes of cell nuclei.

#### 5.3.5. Wound Healing Assay

H1299 cells were seeded in a 6-well plate. When the cell confluency reached 95%, a wound line was scratched using a 200 μl pipette tip and then washed three times. Then, fresh medium containing **4b** or **4c** (2, 4 and 8 μM) was added and the plate was incubated at 37 °C with 5% CO_2_. All images were captured at 0 h and 24 h under an inverted microscope, and the quantification of scratches was analyzed using Image J (https://imagej.net/ij/, accessed on 4 January 2024).

#### 5.3.6. Transwell Migration Assay

Transwell assay was used to determine the migration ability of H1299 cells. Cells were starved in serum-free RPMI 1640 medium for 24 h, then detached and resuspended in serum-free RPMI 1640 medium. 5 × 10^5^ cells/mL were inoculated into the upper chamber of a 24-well Transwell plate (Corning Inc., Corning, NY, USA), with a volume of 100 μL per well. Then, 100 μL serum-free medium containing **4b** or **4c** (2, 4 and 8 μM) was added to the upper chamber and the lower chamber was filled with 700 μL medium containing 20% FBS. After incubation for 24 h, H1299 cells on the upper membrane of the transwell were wiped off. The migrated cells were treated with 4% paraformaldehyde for 20 min, stained with 0.1% crystal violet for 20 min and washed three times with PBS. The number of H1299 cells that migrated to the underside of the membrane was counted under the inverted fluorescence microscope. Three randomly selected areas from each transwell were photographed and calculated using Image J.

#### 5.3.7. Colony Formation Assay

H1299 cells were seeded in six-well plates at a density of 500 cells per well and treated with **4b** or **4c** (1, 2 and 4 μM) for seven days. Then, cells were per-fixed with 4% paraformaldehyde for 30 min, stained with 0.1% crystal violet for 15 min and then washed with pure water. After destain with water, the plates were allowed to dry at room temperature overnight. Image J was used to count the number of clone formations.

#### 5.3.8. Western Blot Analysis

Firstly, H1299 cells were treated with the different concentrations of **4b** and **4c** at 4, 8 and 16 μM for 48 h. Then, cells were harvested using RIPA lysate (R0010, Solarbio, Beijing, China) and centrifuged at 12,000× *g* rpm for 15 min at 4 °C. Following that, the concentration of total protein was measured and taken from the supernatant. Protein was separated by 12% SDS-polyacrylamide gel electrophoresis and transferred to nitrocellulose filter membrane. After blocking in 5% milk for 2 h, the membranes were incubated with a specific primary antibody at 4 °C overnight. The next day, NC membranes were incubated for 1 h at room temperature with appropriate secondary antibodies and then protein bands were visualizing by chemiluminescence detection (ECL kit, Genview, Beijing, China).

### 5.4. Statistical Analyses

We performed statistical analyses using GraphPad Prism 9.5 software. Data were analyzed using one-way analysis of variance (ANOVA) followed by Dunnett’s tests. Data were obtained from no fewer than three independent experiments. A *p*-value less than 0.05 was considered statistically significant.

## Figures and Tables

**Table 1:** IC_50_ values of test compounds against NCI-H1299, A549 and NCI-H1437 cell lines.

Compd No.	IC_50_ (μM)		
	H1299	A549	NCI-H1437
**4a**	7.59 ± 0.19	6.81 ± 0.33	2.05 ± 0.03
**4b**	**4.42 ± 0.24**	**3.94 ± 0.01**	**1.56 ± 0.06**
**4c**	**4.60 ± 0.18**	**4.00 ± 0.08**	**3.51 ± 0.05**
**4d**	11.44 ± 0.97	9.04 ± 0.41	6.41 ± 0.54
**4e**	11.88 ± 0.32	8.62 ± 0.76	11.16 ± 0.95
**4f**	13.26 ± 0.52	13.65 ± 0.71	9.27 ± 0.40
**4g**	6.02 ± 0.12	4.65 ± 0.08	2.15 ± 0.10
**4h**	7.69 ± 0.11	9.81 ± 0.34	3.09 ± 0.13
**4i**	9.35 ± 0.31	12.96 ± 0.29	4.77 ± 0.32
**4j**	12.02 ± 0.33	11.41 ± 0.89	6.60 ± 0.07
**4k**	7.15 ± 0.12	8.95 ± 0.99	5.95 ± 0.72
**4l**	5.43 ± 0.24	7.53 ± 0.66	2.23 ± 0.26
**4m**	4.79 ± 0.13	2.39 ± 0.30	2.53 ± 0.11
**4n**	5.91 ± 0.44	10.41 ± 0.82	5.79 ± 0.88
Gefitinib ^a^	14.23 ± 0.08	15.11 ± 0.05	20.44 ± 1.43

The bold values represent the IC_50_ values of **4b** and **4c** with significant inhibitory effects. All values are given as means ± standard deviation. (*n* = 3) ^a^ Gefitinib was used as positive control in this study.

**Table 2:** Inhibitory rate of **4b** and **4c** in L02 cells.

Compound No.	IC_50_ (μM)	Inhibition Rate %, 48 h				
		2 μM	4 μM	8 μM	16 μM	32 μM
**4b**	20.25 ± 1.26	9.09 ± 1.00	12.76 ± 1.37	29.76 ± 1.27	39.08 ± 2.18	64.85 ± 1.84
**4c**	29.38 ± 5.53	7.18 ± 1.22	20.72 ± 3.30	27.55 ± 2.79	30.56 ± 1.93	56.13 ± 5.77

All values are given as means ± standard deviation (*n* = 3).

## Data Availability

The datasets used and/or analyzed during the current study are available from the corresponding authors upon reasonable request.

## Supplementary Materials

The following supporting information can be downloaded at: https://www.mdpi.com/article/10.3390/molecules29040837/s1, The nuclear magnetic resonance spectroscopy (^1^H NMR and ^13^C NMR) and high-resolution mass spectrometry (HRMS) of compounds **4a–4n** are Supplementary Materials provided as data.
