# Supplementary material for: Synthesis and In Vitro Antitumor Activity Evaluation of Gefitinib-1,2,3-Triazole Derivatives
Source: Molecules. 2024 Feb 13;29(4):837. doi: 10.3390/molecules29040837 (PMC10892142; doi:10.3390/molecules29040837)

Supplementary Information

Synthesis and In Vitro Antitumor Activity Evaluation of Gefitinib-1,2,3-triazole Derivatives

Zijun Liu[1], Jiancheng Liu[1], En Gao[2], Longfei Mao[1*] Shu Hu[1*] Sanqiang Li[1*]

[1] College of Basic Medicine and Forensic Medicine, Henan University of Science and Technology, Luoyang 471023, China.

[2]School of Chemistry and Chemical Engineering, Henan Normal University, Xinxiang, 453007, China

*Corresponding authors:

Sanqiang Li, College of Basic Medicine and Forensic Medicine, Henan University of Science and Technology, Luoyang 471023, China.

E-mail: sanqiangli2001@163.com

Shu Hu, College of Basic Medicine and Forensic Medicine, Henan University of Science and Technology, Luoyang 471023, China.

E-mail: shuhu20102023@163.com

Longfei Mao, College of Basic Medicine and Forensic Medicine, Henan University of Science and Technology, Luoyang 471023, China.

E-mail: longfeimao1988@163.com

Table of Content

Figure S1-1. ^{1}H NMR spectrum (400 MHz, DMSO-d_6) of compound 4a 1

Figure S1-2. ^{13}C NMR spectrum (100 MHz, DMSO-d_6) of compound 4a........................ 2

Figure S1-3. HR MS of compound 4a.. 2

Figure S2-1. ^{1}H NMR spectrum (400 MHz, DMSO-d_6) of compound 4b 3

Figure S2-2. ^{13}C NMR spectrum (100 MHz, DMSO-d_6) of compound 4b 4

Figure S2-3. HR MS of compound 4b 4

Figure S3-1. ^{1}H NMR spectrum (400 MHz, DMSO-d_6) of compound 4c......................... 5

Figure S3-2. ^{13}C NMR spectrum (100 MHz, DMSO-d_6) of compound 4c 6

Figure S3-3. HR MS of compound 4c.. 6

Figure S4-1. ^{1}H NMR spectrum (400 MHz, Methanol-d_4) of compound 4d 7

Figure S4-2. ^{13}C NMR spectrum (100 MHz, Methanol-d_4) of compound 4d 8

Figure S4-3. HR MS of compound 4d 8

Figure S5-1. ^{1}H NMR spectrum (400 MHz, DMSO-d_6) of compound 4e......................... 9

Figure S5-2. ^{13}C NMR spectrum (100 MHz, DMSO-d_6) of compound 4e...................... 10

Figure S5-3. HR MS of compound 4e.. 10

Figure S6-1. ^{1}H NMR spectrum (400 MHz, DMSO-d_6) of compound 4f....................... 11

Figure S6-2. ^{13}C NMR spectrum (100 MHz, DMSO-d_6) of compound 4f 12

Figure S6-3. HR MS of compound 4f 12

Figure S7-1. ^{1}H NMR spectrum (400 MHz, DMSO-d_6) of compound 4g 13

Figure S7-2. ^{13}C NMR spectrum (100 MHz, DMSO-d_6) of compound 4g 14

Figure S7-3. HR MS of compound 4g 14

Figure S8-1. ^{1}H NMR spectrum (400 MHz, DMSO-d_6) of compound 4h 15

Figure S8-2. ^{13}C NMR spectrum (100 MHz, DMSO-d_6) of compound 4h 16

Figure S8-3. HR MS of compound 4h 16

Figure S9-1. ^{1}H NMR spectrum (400 MHz, DMSO-d_6) of compound 4i 17

Figure S9-2. ^{13}C NMR spectrum (100 MHz, DMSO-d_6) of compound 4i....................... 18

Figure S9-3. HR MS of compound 4i 18
Figure S10-1. ^{1}H NMR spectrum (400 MHz, DMSO-d_6) of compound 4j 19
Figure S10-2. ^{13}C NMR spectrum (100 MHz, DMSO-d_6) of compound 4j 20
Figure S10-3. HR MS of compound 4j 20
Figure S11-1. ^{1}H NMR spectrum (400 MHz, DMSO-d_6) of compound 4k 21
Figure S11-2. ^{13}C NMR spectrum (100 MHz, DMSO-d_6) of compound 4k 22
Figure S11-3. HR MS of compound 4k 22
Figure S12-1. ^{1}H NMR spectrum (400 MHz, DMSO-d_6) of compound 4l 23
Figure S12-2. ^{13}C NMR spectrum (100 MHz, DMSO-d_6) of compound 4l 24
Figure S12-3. HR MS of compound 4l 24
Figure S13-1. ^{1}H NMR spectrum (400 MHz, DMSO-d_6) of compound 4m 25
Figure S13-2. ^{13}C NMR spectrum (100 MHz, DMSO-d_6) of compound 4m 26
Figure S13-3. HR MS of compound 4m 26
Figure S14-1. ^{1}H NMR spectrum (400 MHz, DMSO-d_6) of compound 4n 27
Figure S14-2. ^{13}C NMR spectrum (100 MHz, DMSO-d_6) of compound 4n 28
Figure S14-3. HR MS of compound 4n 28

Figure S1-1. ^{1}H NMR spectrum (400 MHz, DMSO-d_6) of compound **4a**

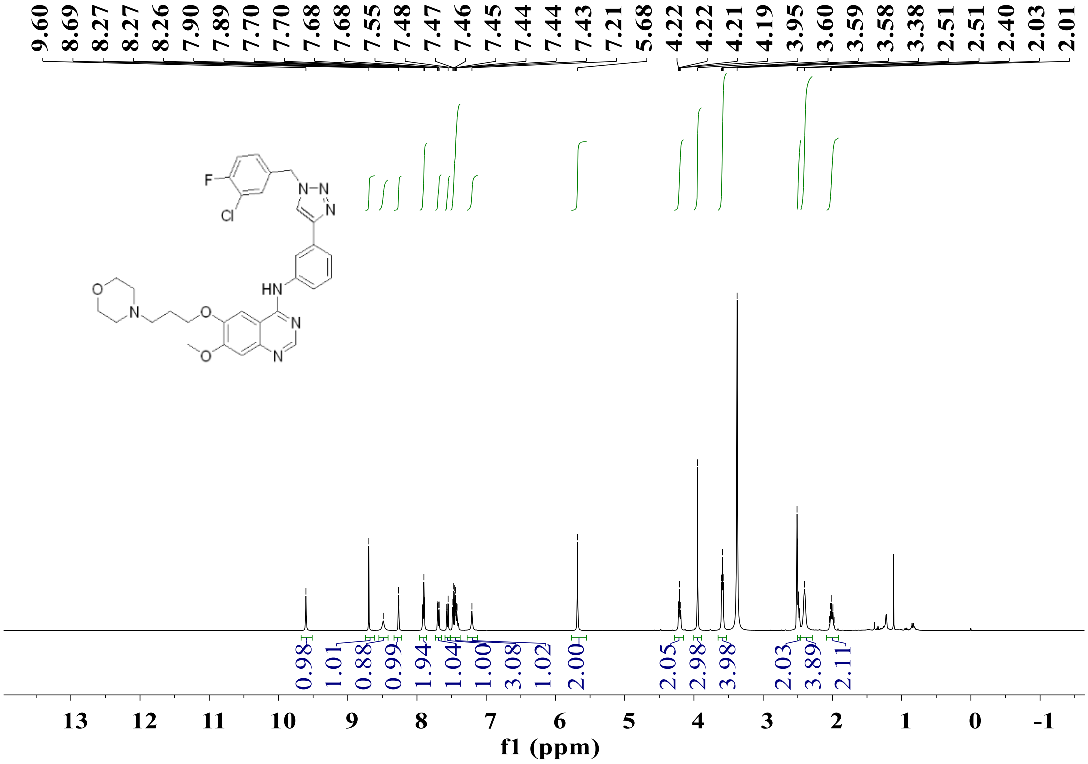

Figure S1-2. ^{13}C NMR spectrum (100 MHz, DMSO-d_6) of compound **4a**

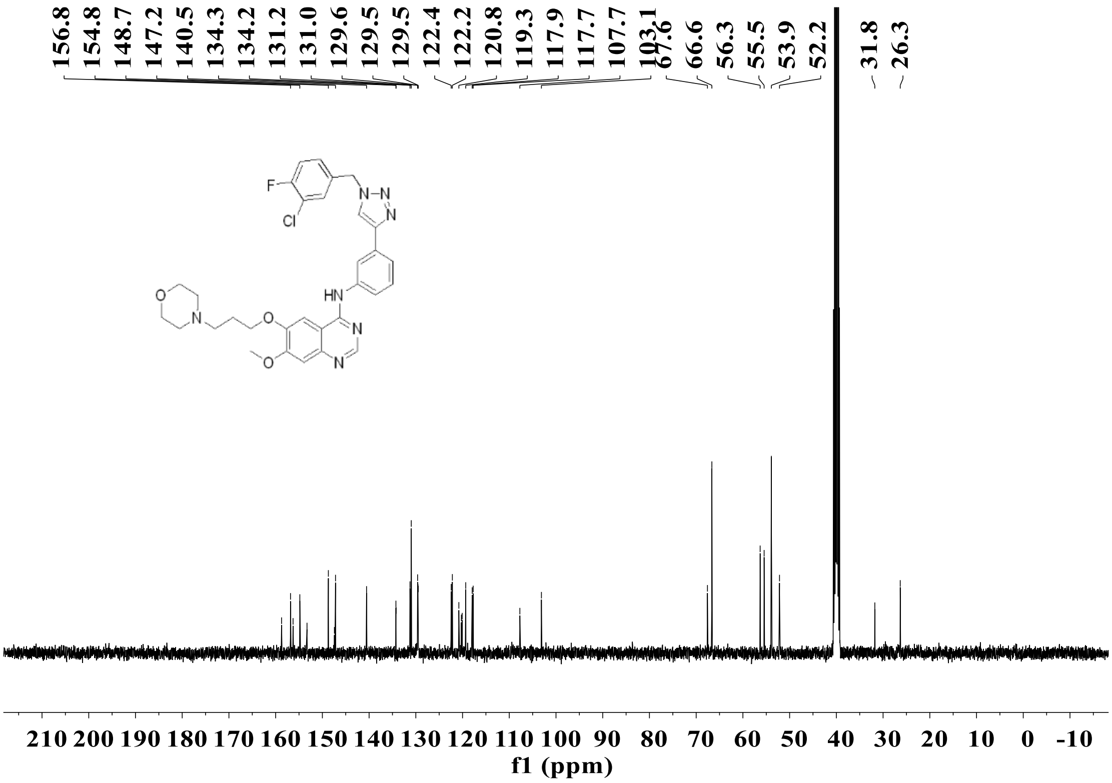

Figure S1-3. HR MS of compound **4a**

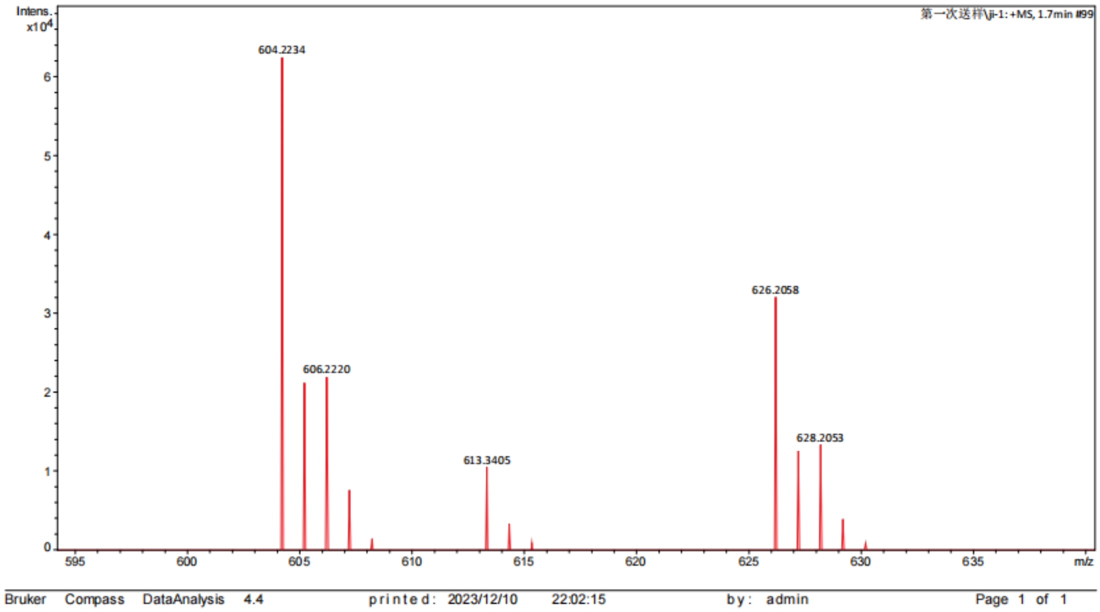

Figure S2-1. ^{1}H NMR spectrum (400 MHz, DMSO-d_6) of compound **4b**

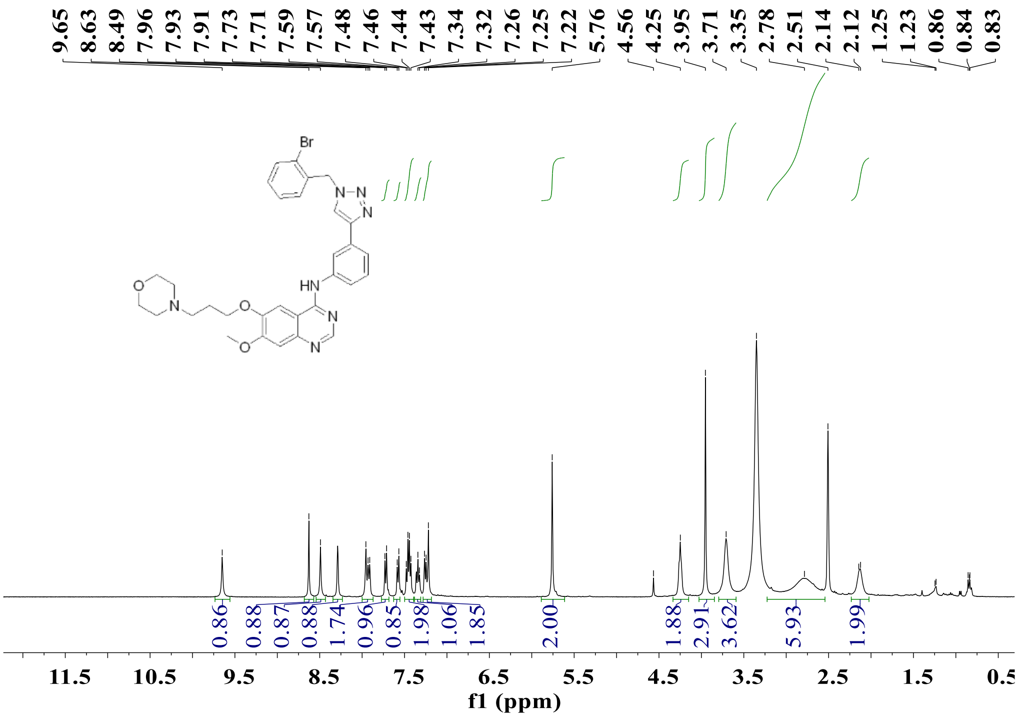

Figure S2-2. ^{13}C NMR spectrum (100 MHz, DMSO-d_6) of compound **4b**

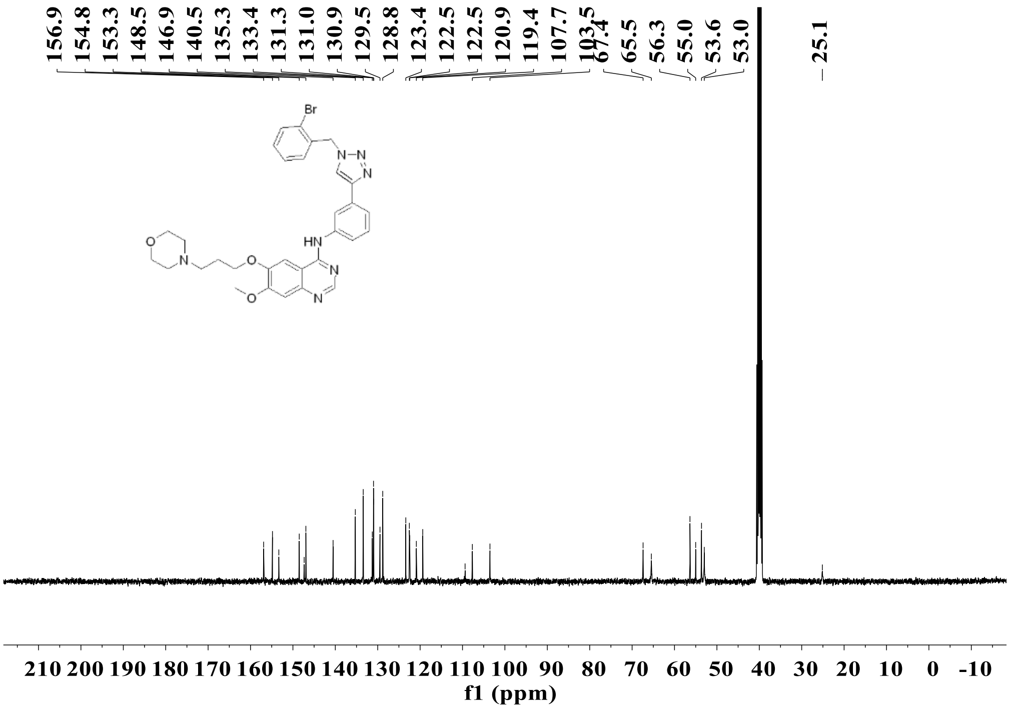

Figure S2-3. HR MS of compound **4b**

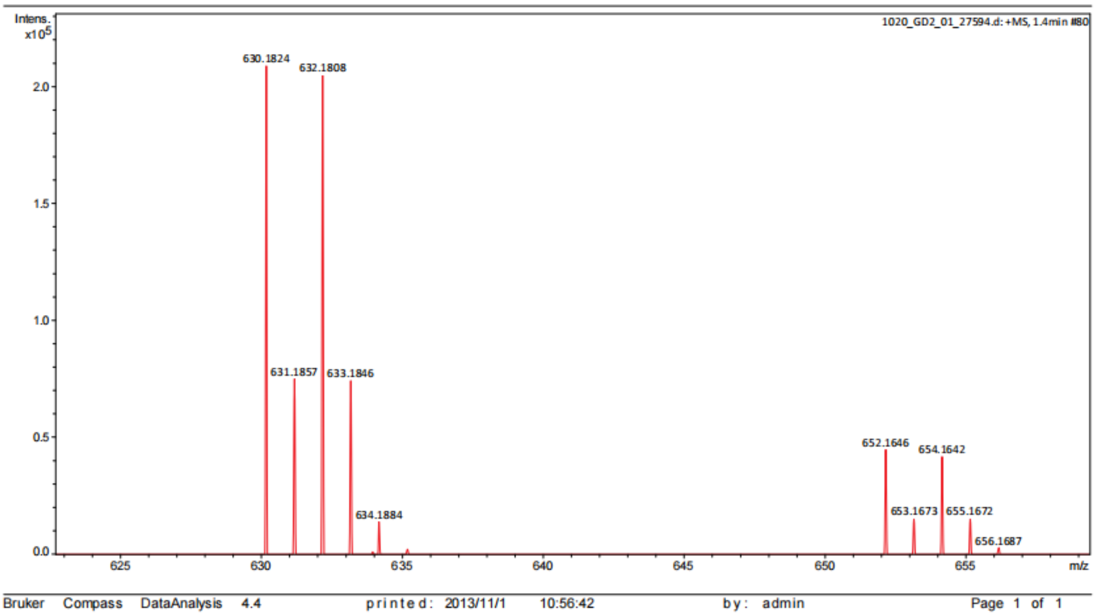

Figure S3-1. ^{1}H NMR spectrum (400 MHz, DMSO-d_6) of compound **4c**

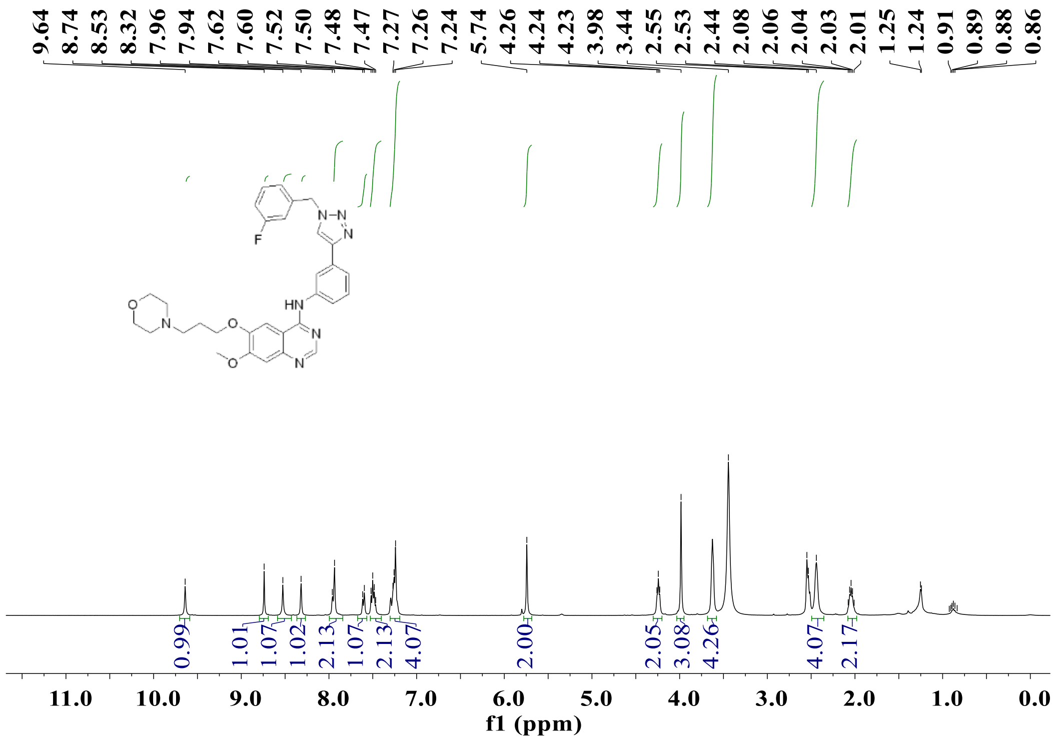

Figure S3-2. ^{13}C NMR spectrum (100 MHz, DMSO-d_6) of compound **4c**

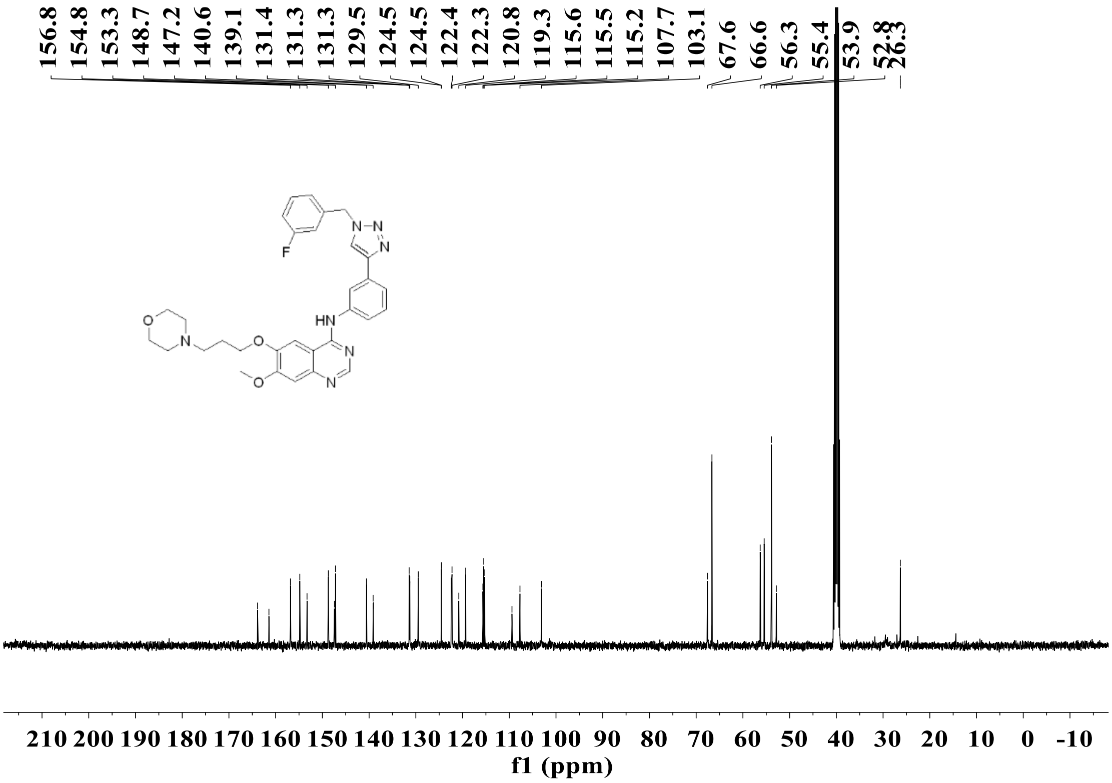

Figure S3-3. HR MS of compound **4c**

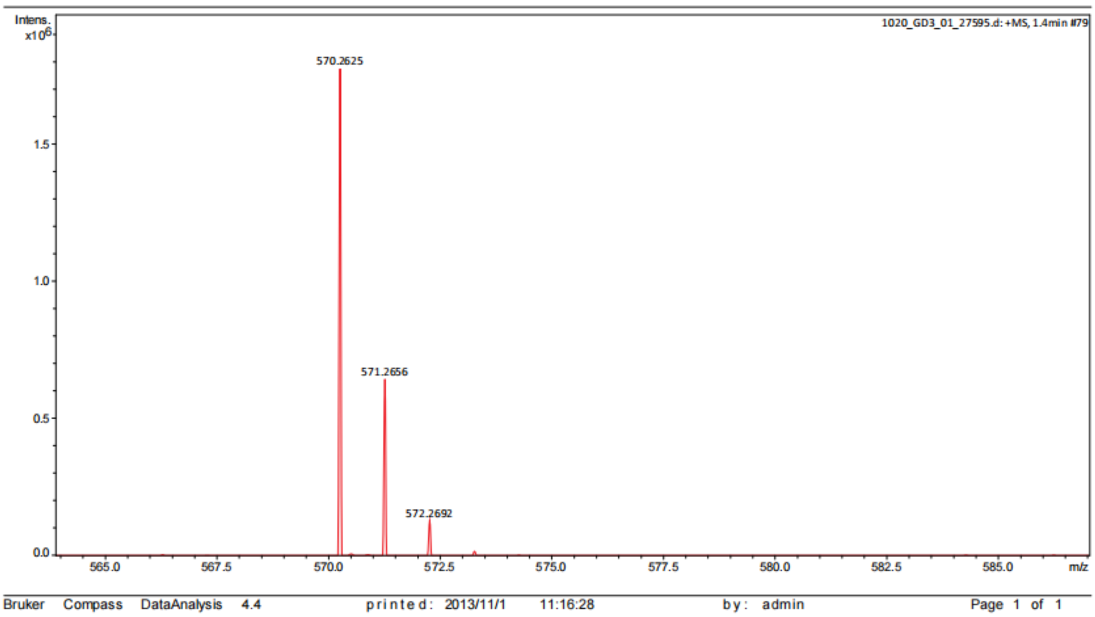

Figure S4-1. ^{1}H NMR spectrum (400 MHz, Methanol-d_4) of compound **4d**

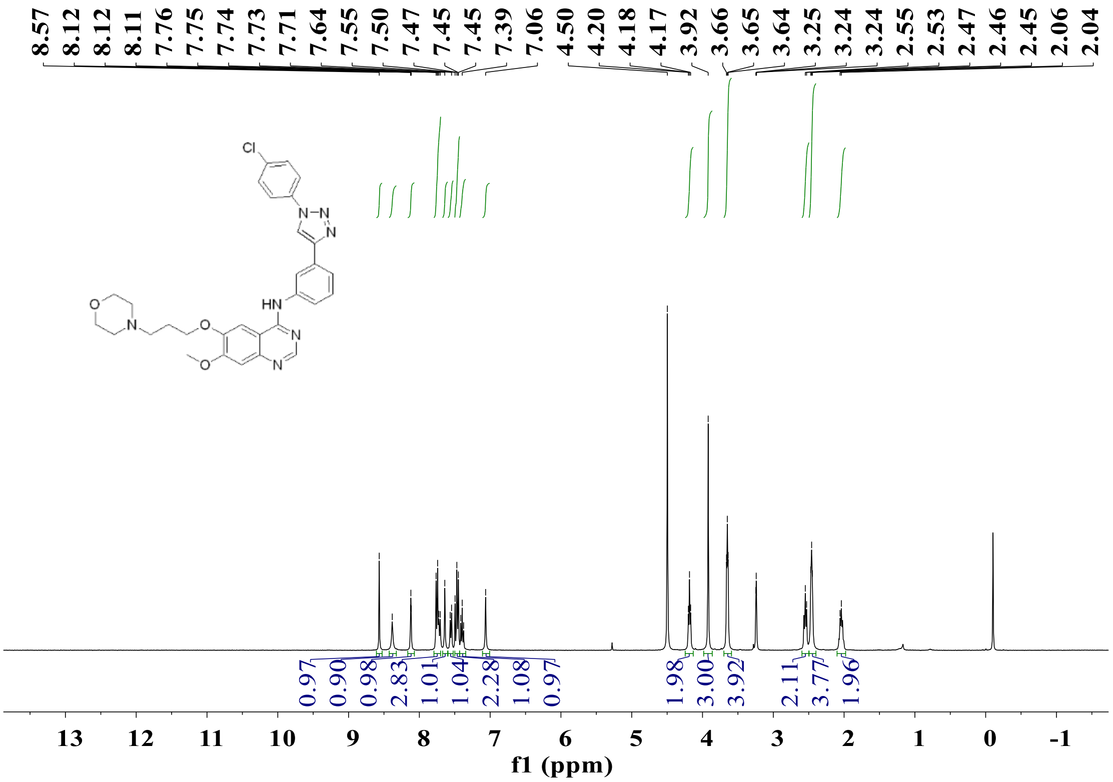

Figure S4-2. ^{13}C NMR spectrum (100 MHz, Methanol-d_4) of compound **4d**

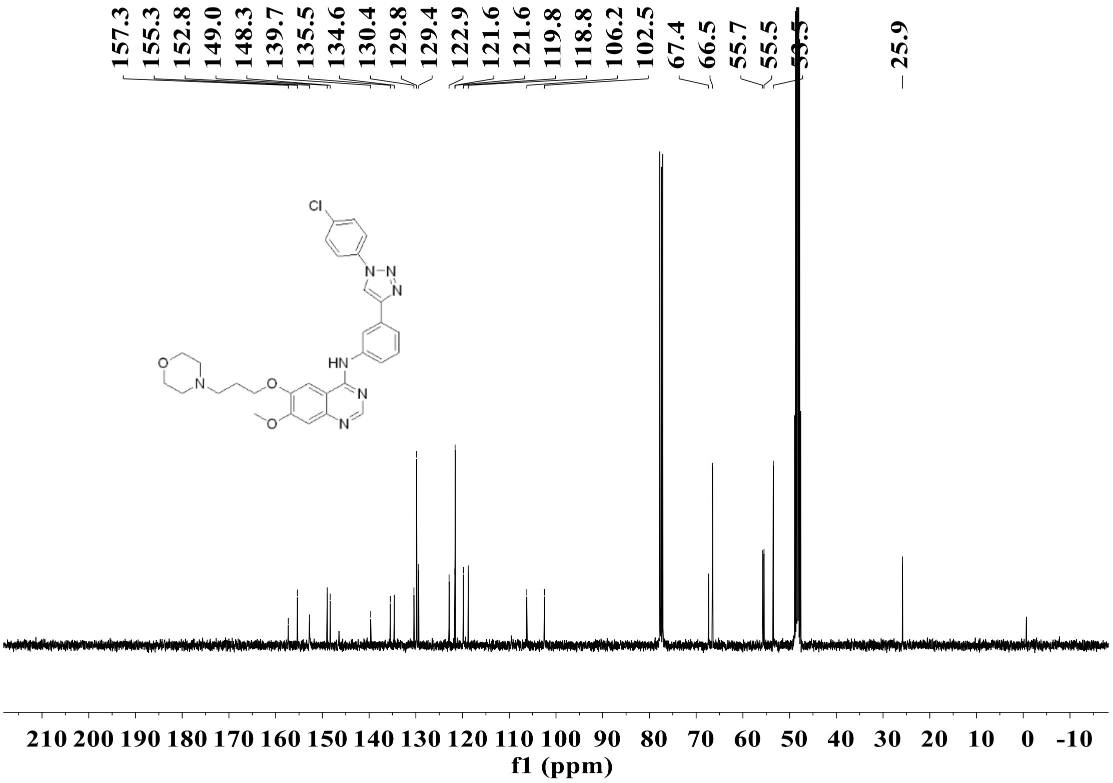

Figure S4-3. HR MS of compound **4d**

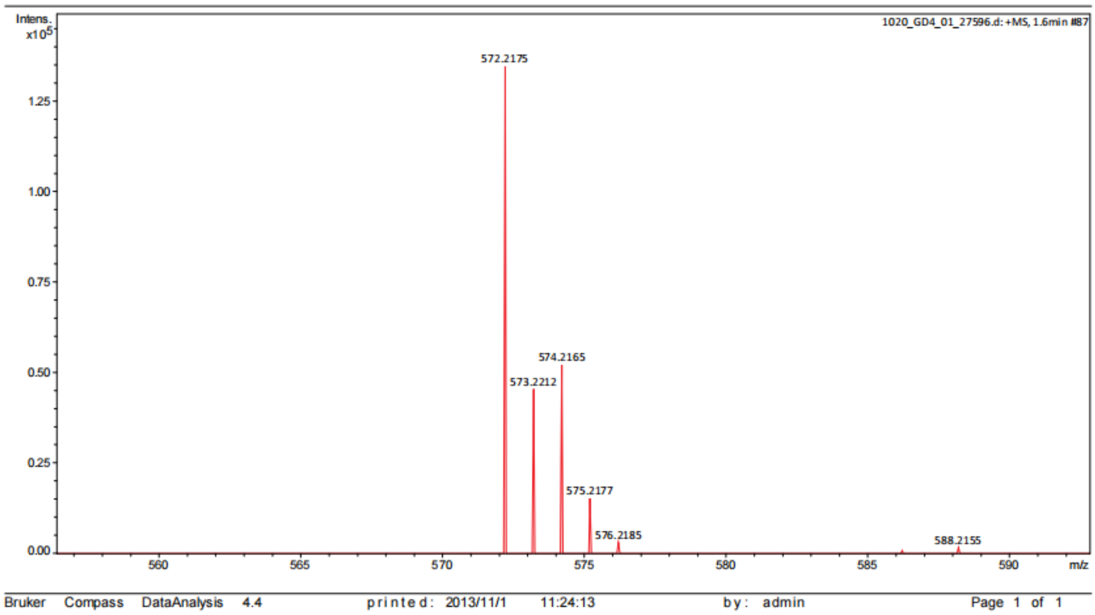

Figure S5-1. ^{1}H NMR spectrum (400 MHz, DMSO-d_6) of compound **4e**

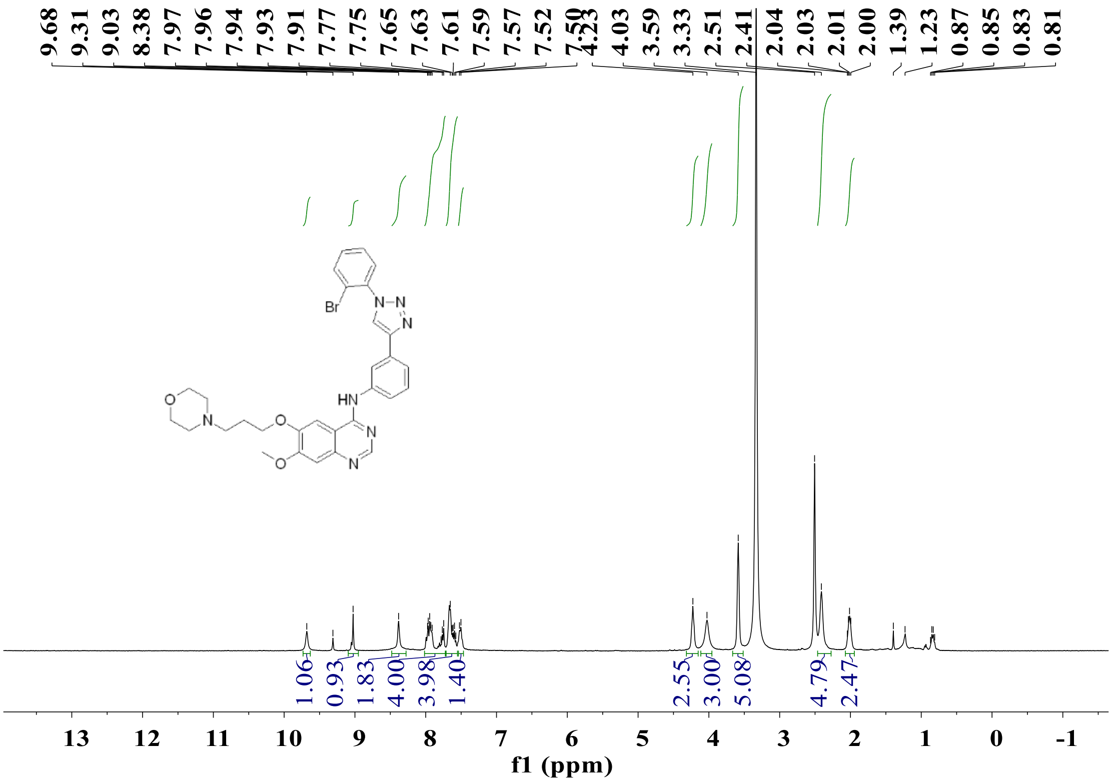

Figure S5-2. ^{13}C NMR spectrum (100 MHz, DMSO-d_6) of compound **4e**

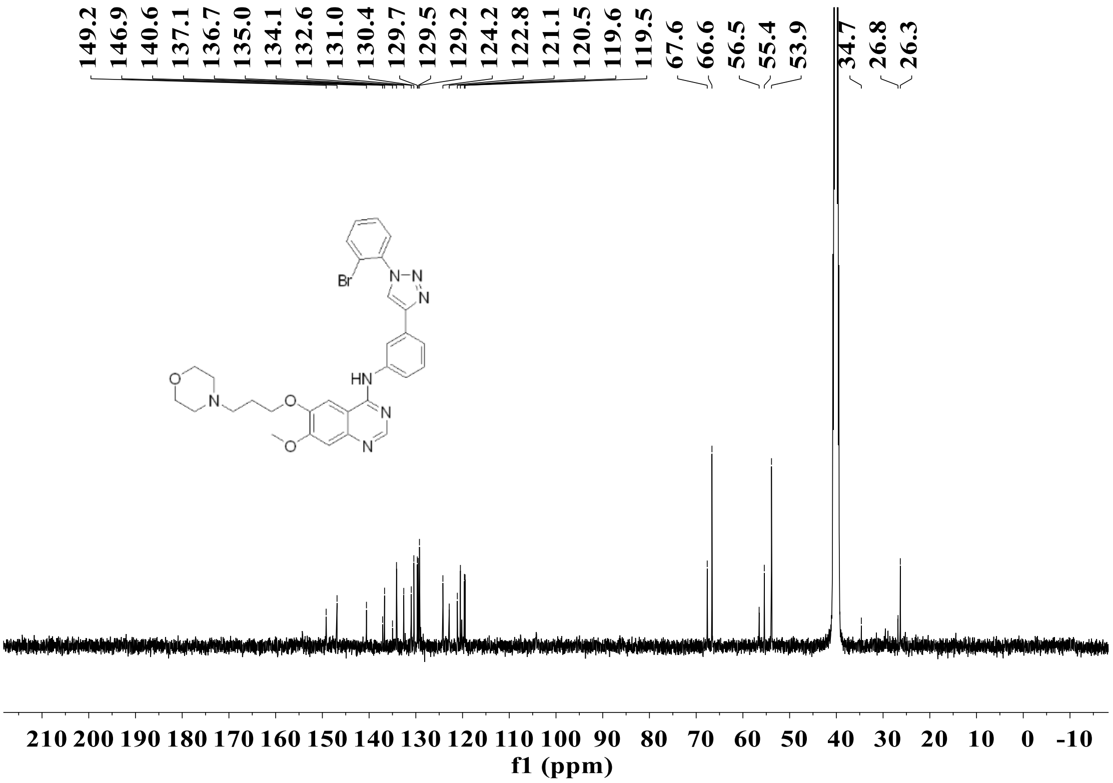

Figure S5-3. HR MS of compound **4e**

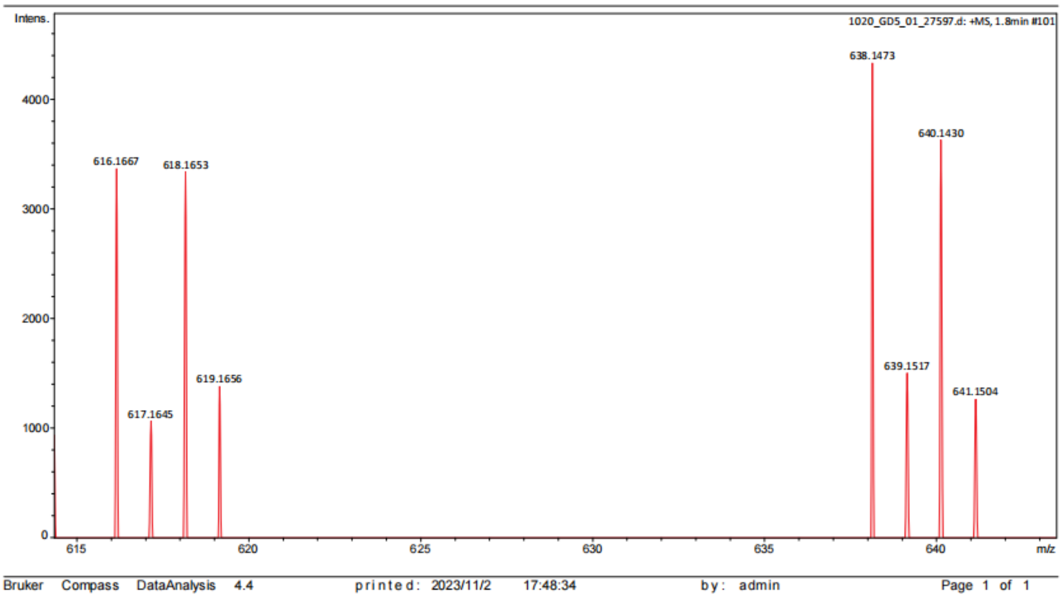

Figure S6-1. ^{1}H NMR spectrum (400 MHz, DMSO-d_6) of compound **4f**

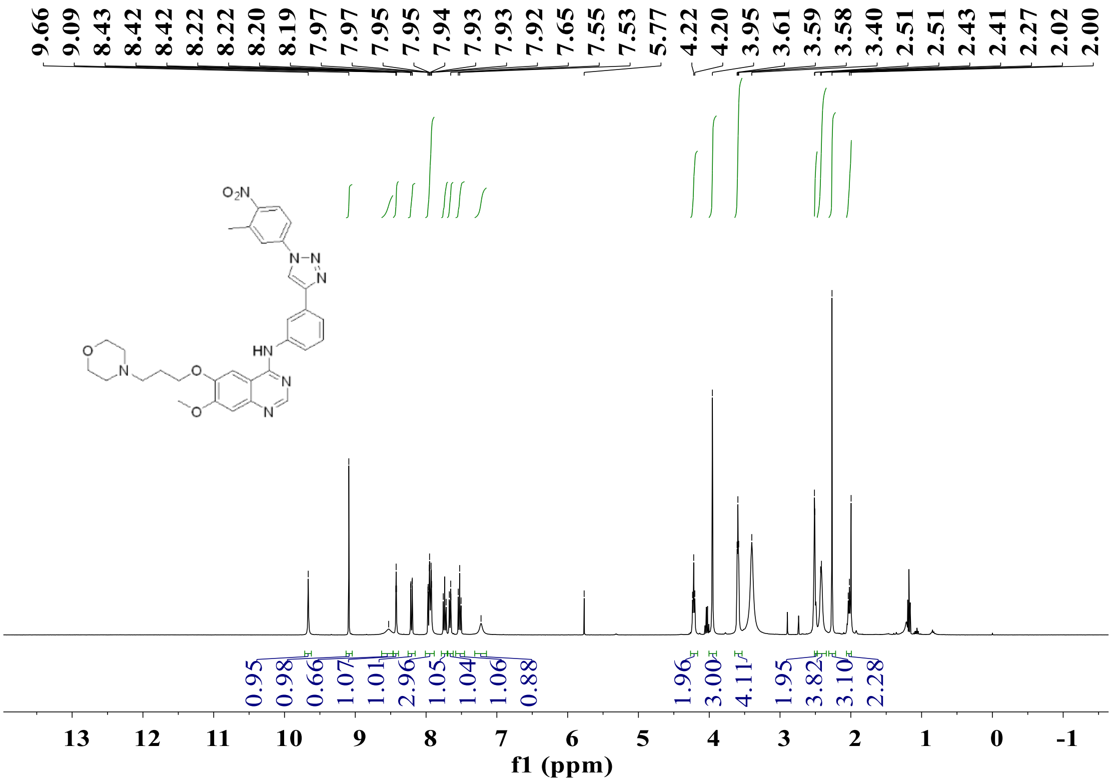

Figure S6-2. ^{13}C NMR spectrum (100 MHz, DMSO-d_6) of compound **4f**

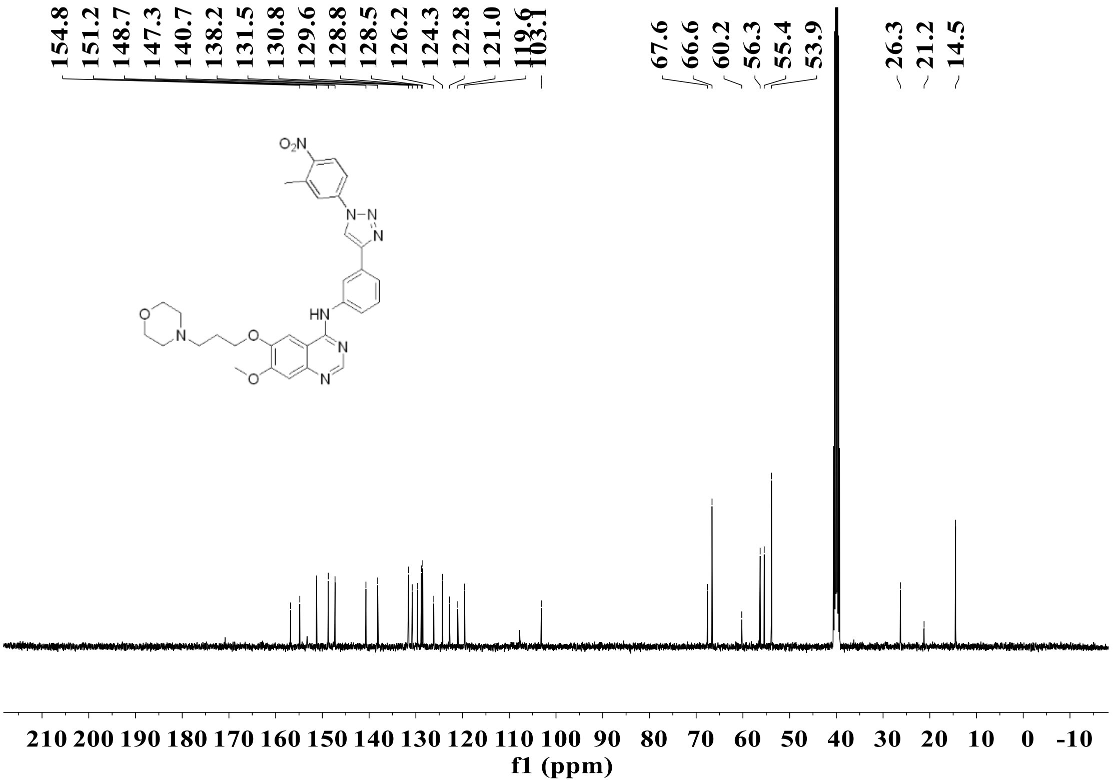

Figure S6-3. HR MS of compound **4f**

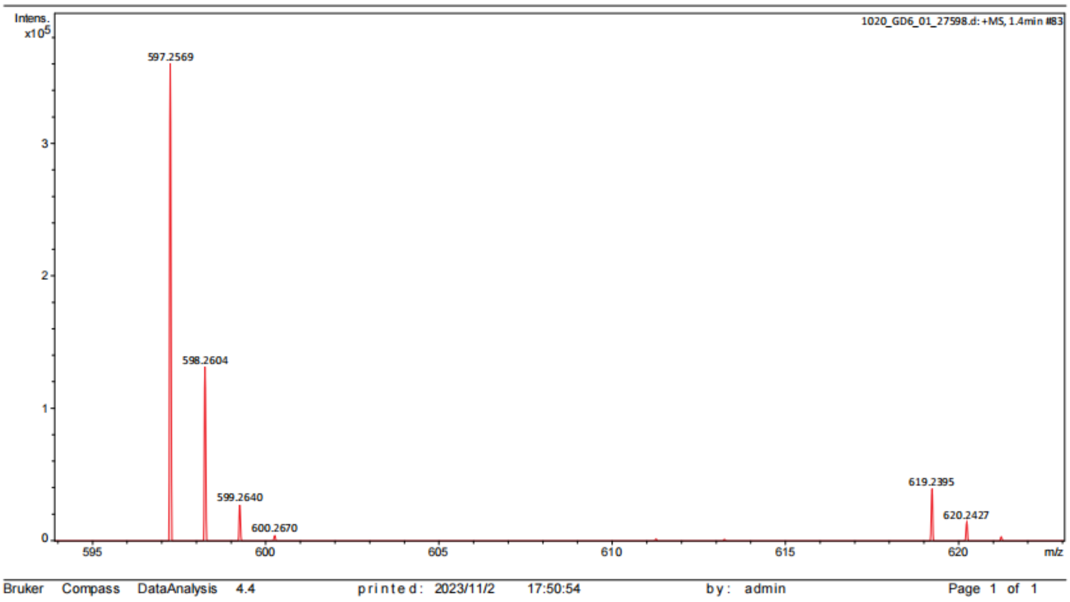

Figure S7-1. ^{1}H NMR spectrum (400 MHz, DMSO-d_6) of compound **4g**

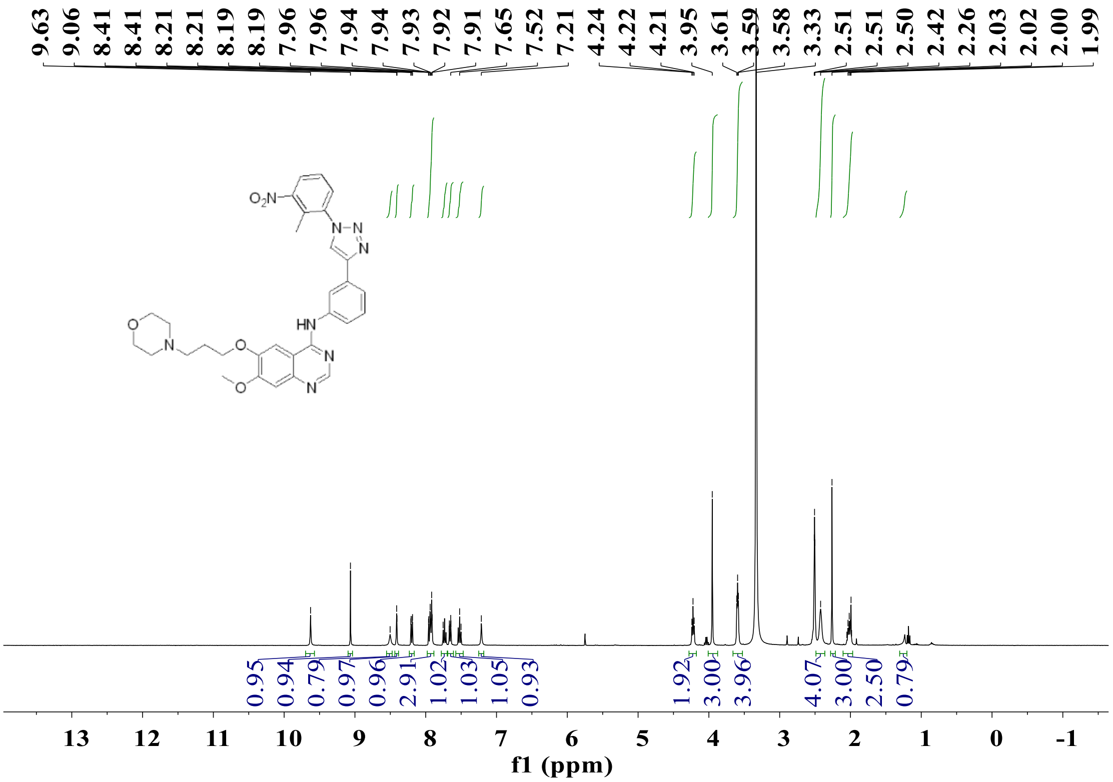

Figure S7-2. ^{13}C NMR spectrum (100 MHz, DMSO-d_6) of compound **4g**

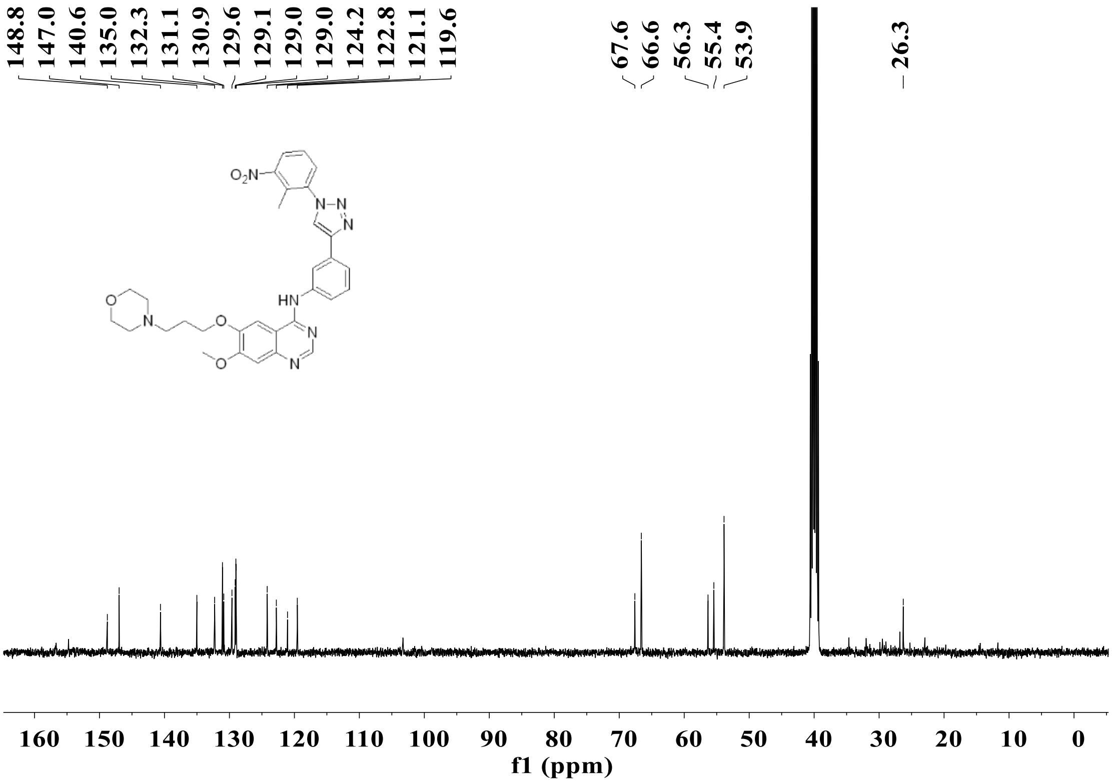

Figure S7-3. HR MS of compound **4g**

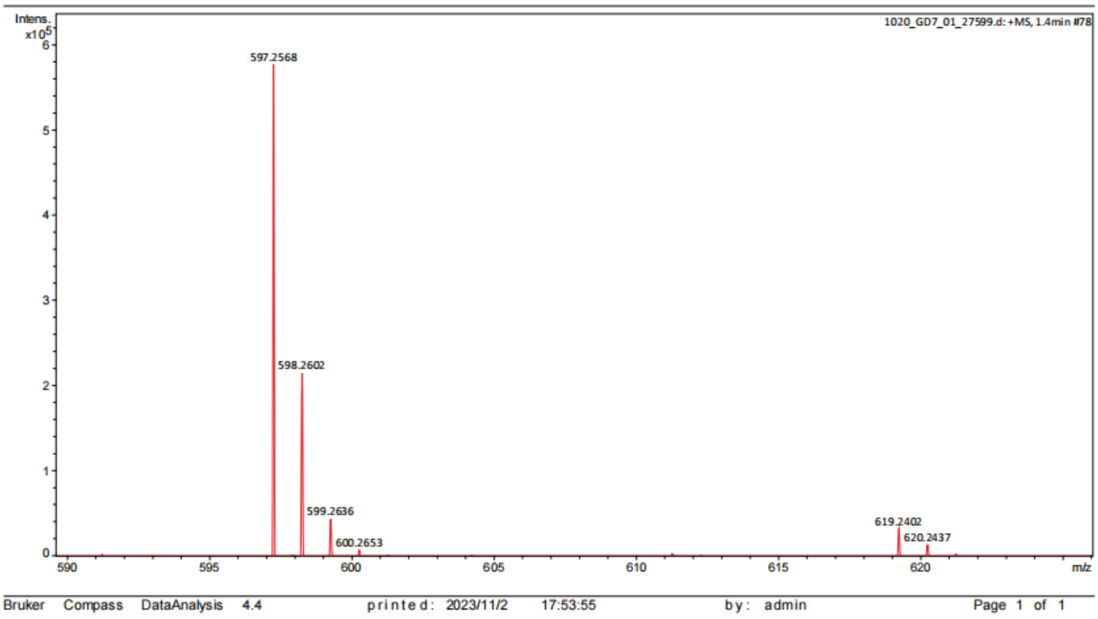

Figure S8-1. ^{1}H NMR spectrum (400 MHz, DMSO-d_6) of compound **4h**

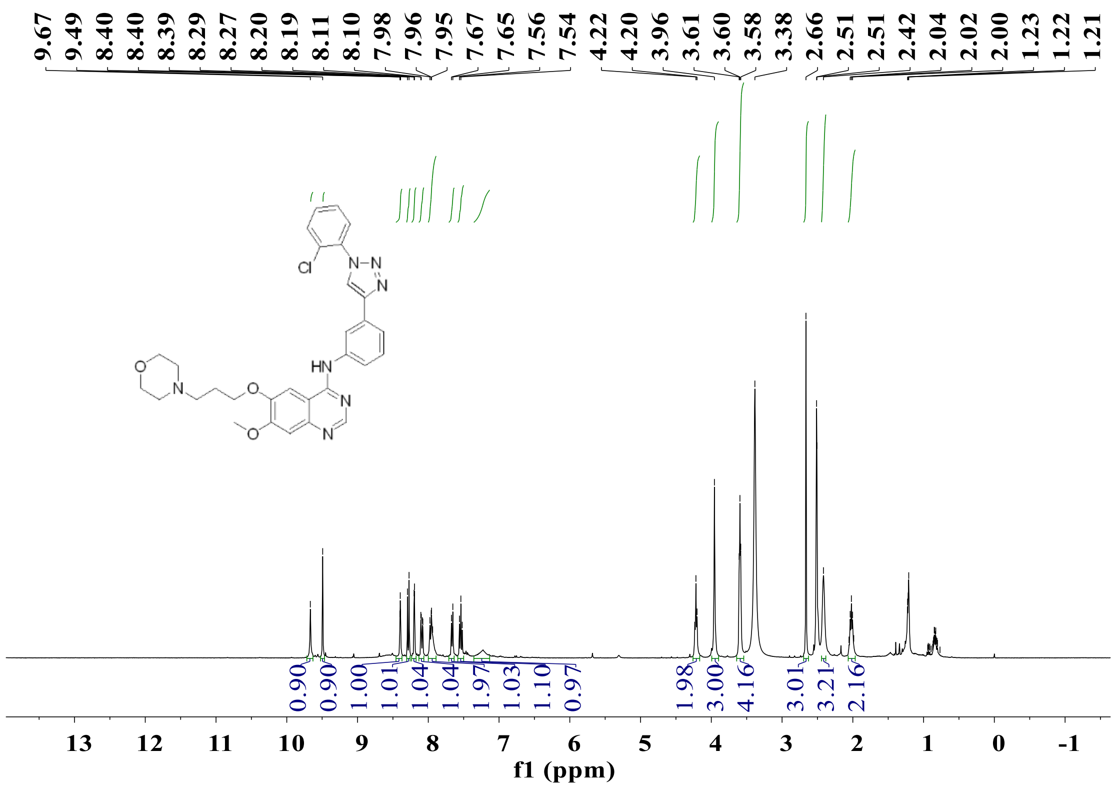

Figure S8-2. ^{13}C NMR spectrum (100 MHz, DMSO-d_6) of compound **4h**

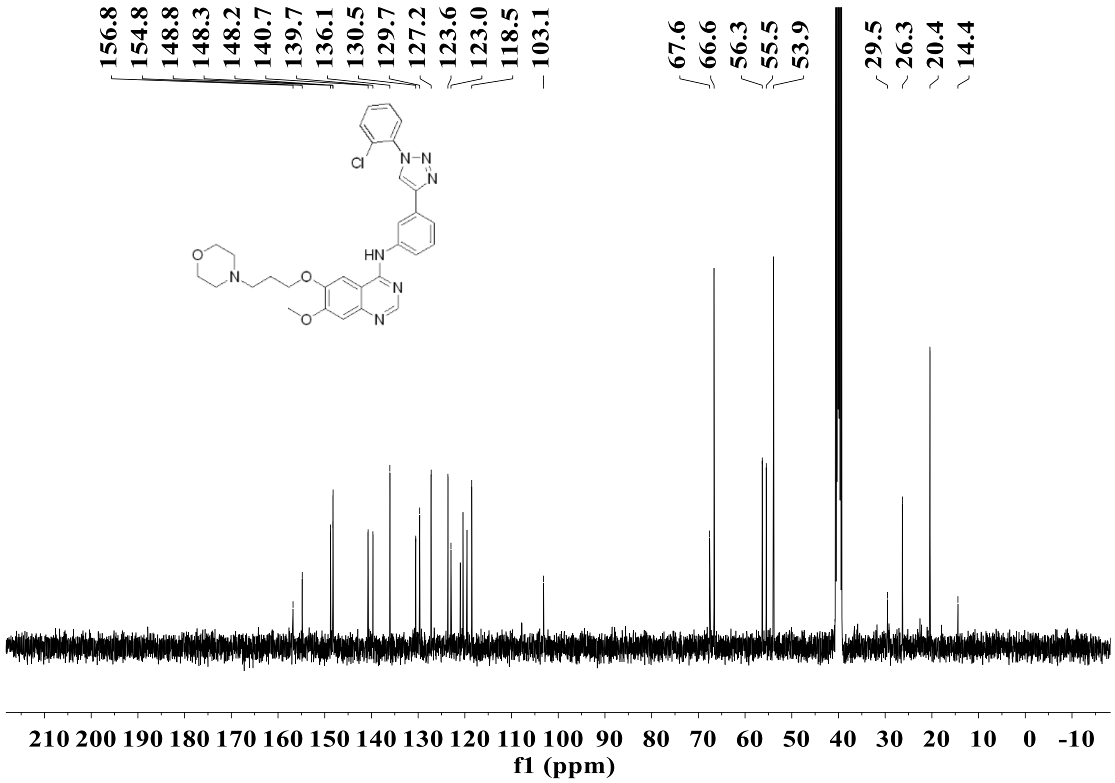

Figure S8-3. HR MS of compound **4h**

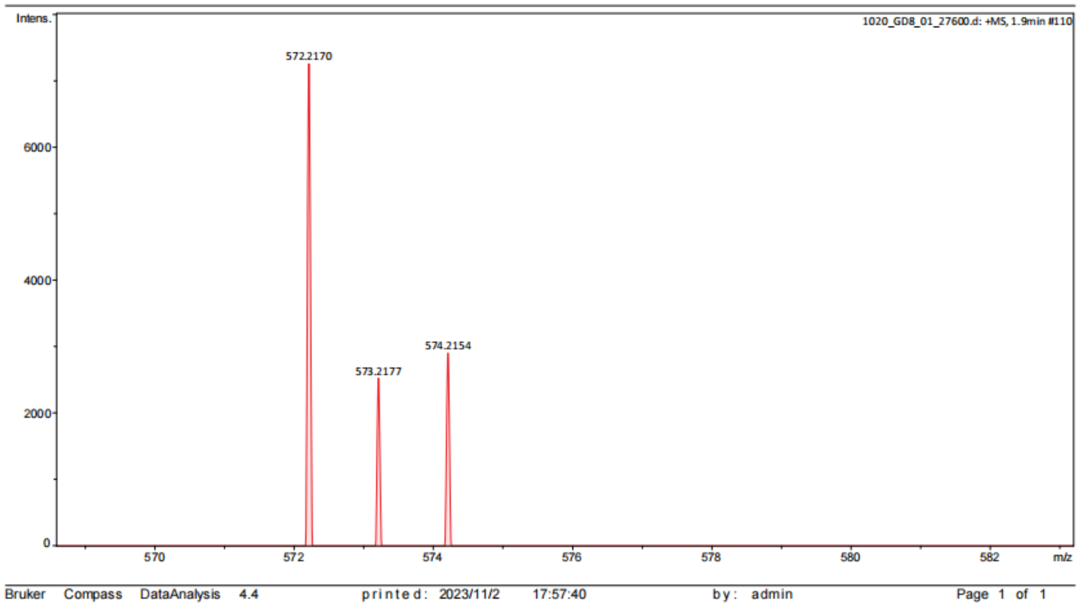

Figure S9-1. ^{1}H NMR spectrum (400 MHz, DMSO-d_6) of compound **4i**

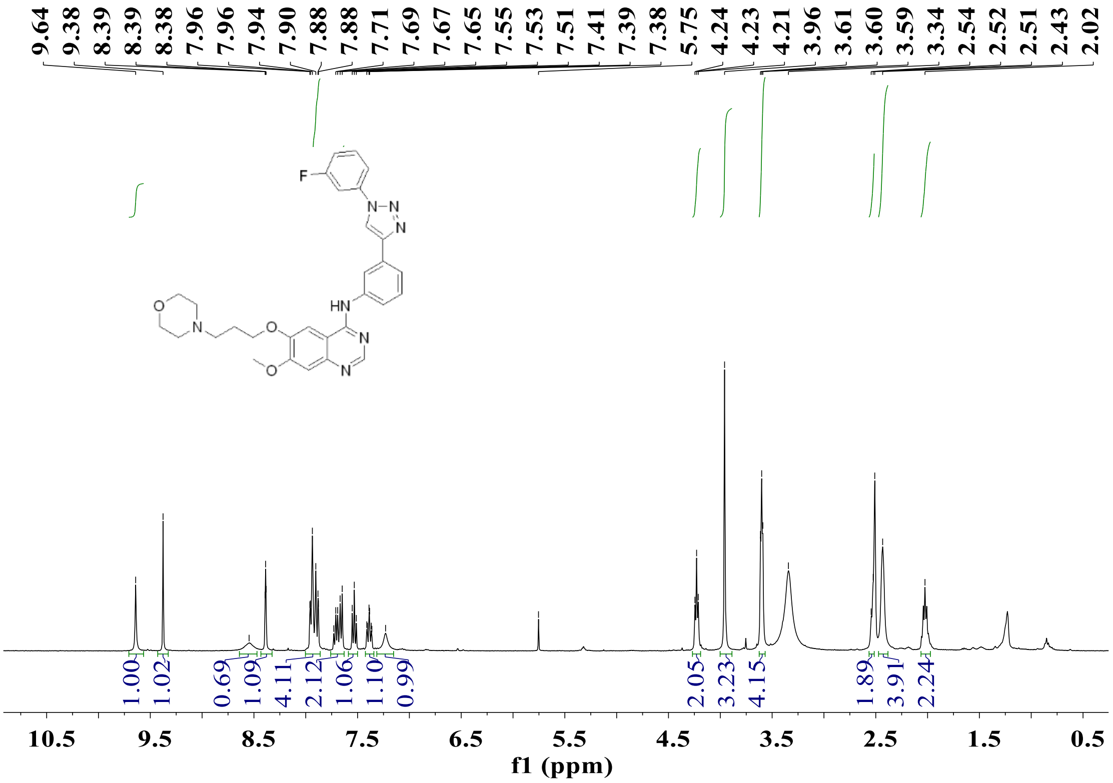

Figure S9-2. ^{13}C NMR spectrum (100 MHz, DMSO-d_6) of compound **4i**

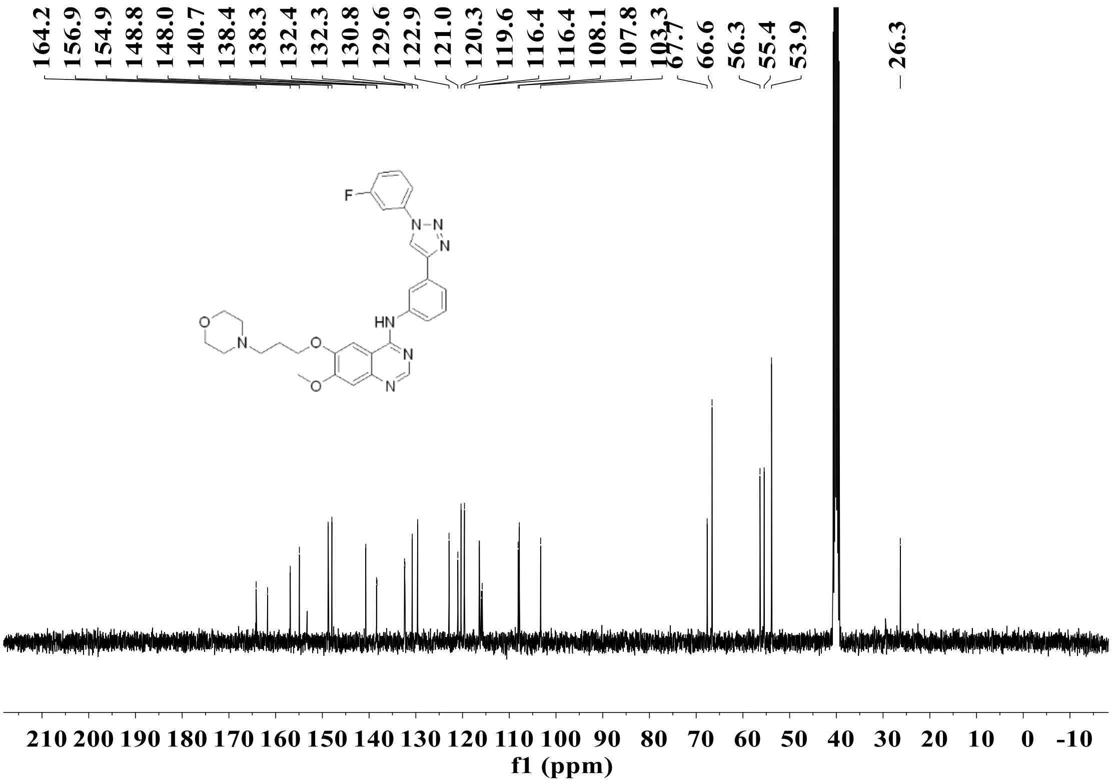

Figure S9-3. HR MS of compound **4i**

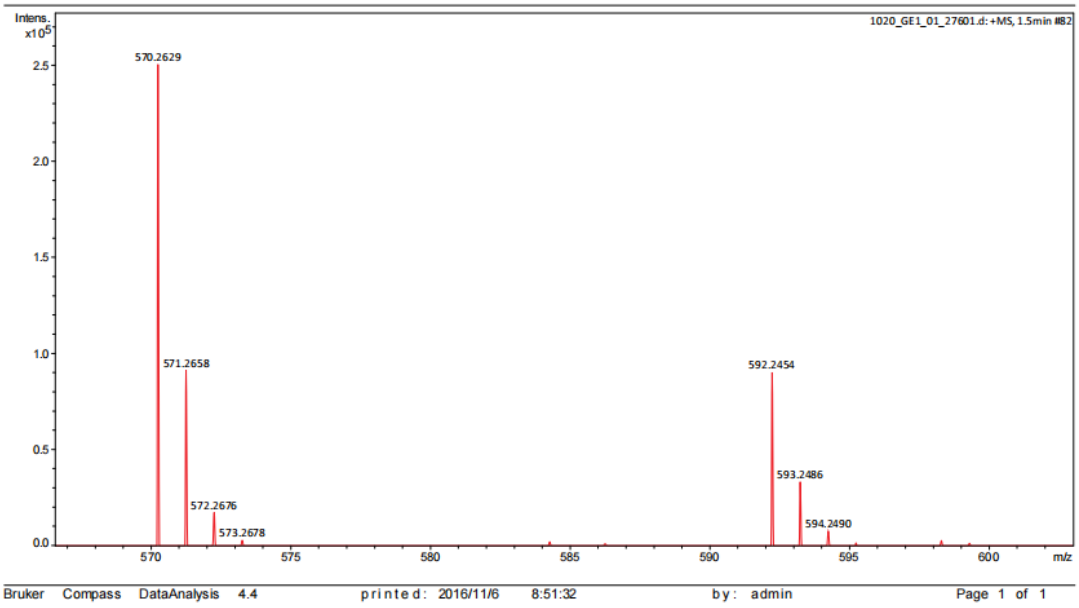

Figure S10-1. ^{1}H NMR spectrum (400 MHz, DMSO-d_6) of compound **4j**

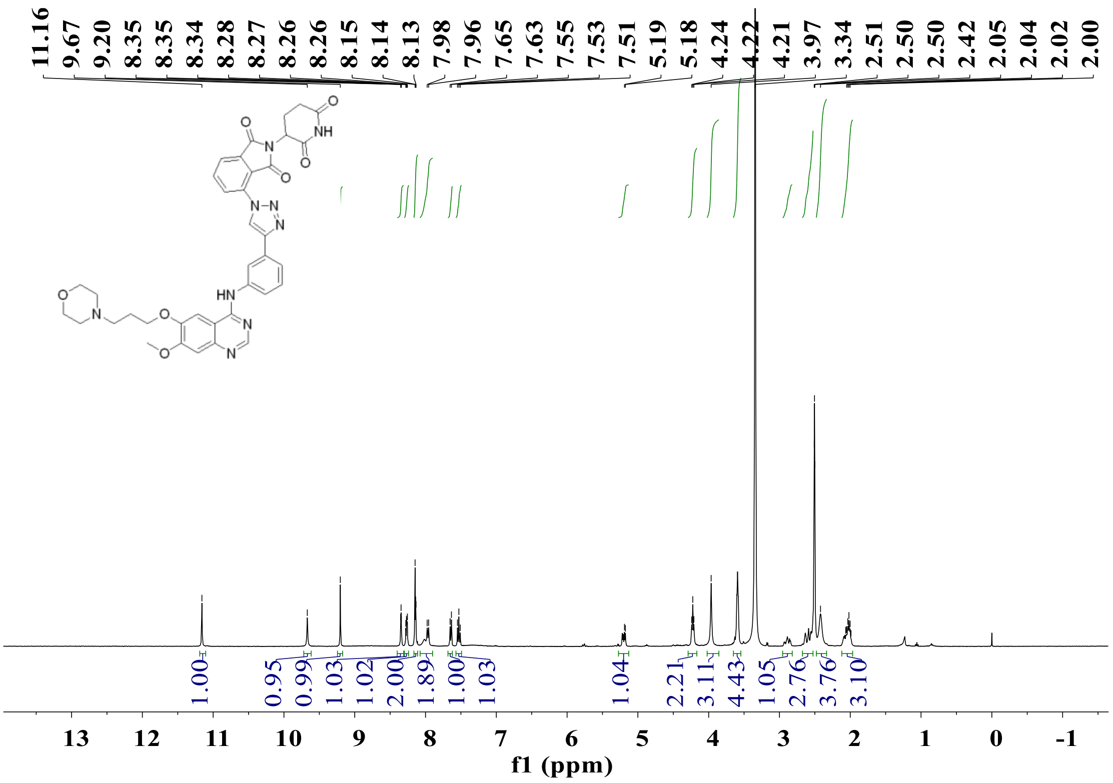

Figure S10-2. ^{13}C NMR spectrum (100 MHz, DMSO-*d*$_6$) of compound **4j**

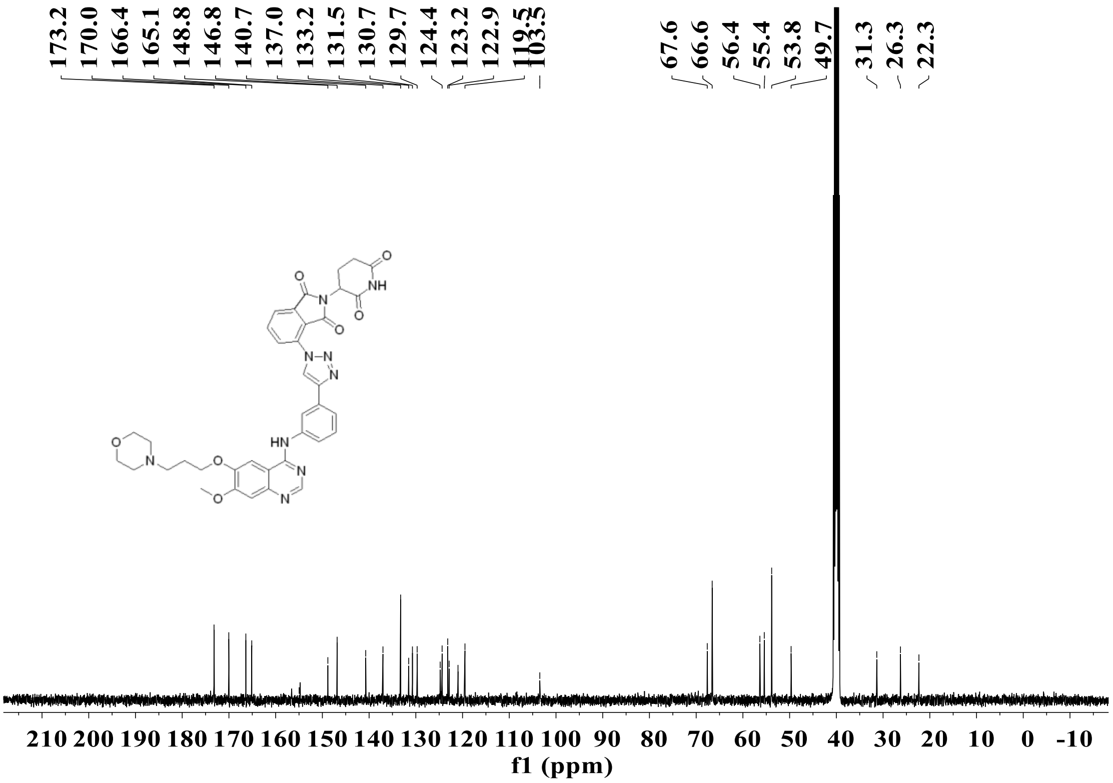

Figure S10-3. HR MS of compound **4j**

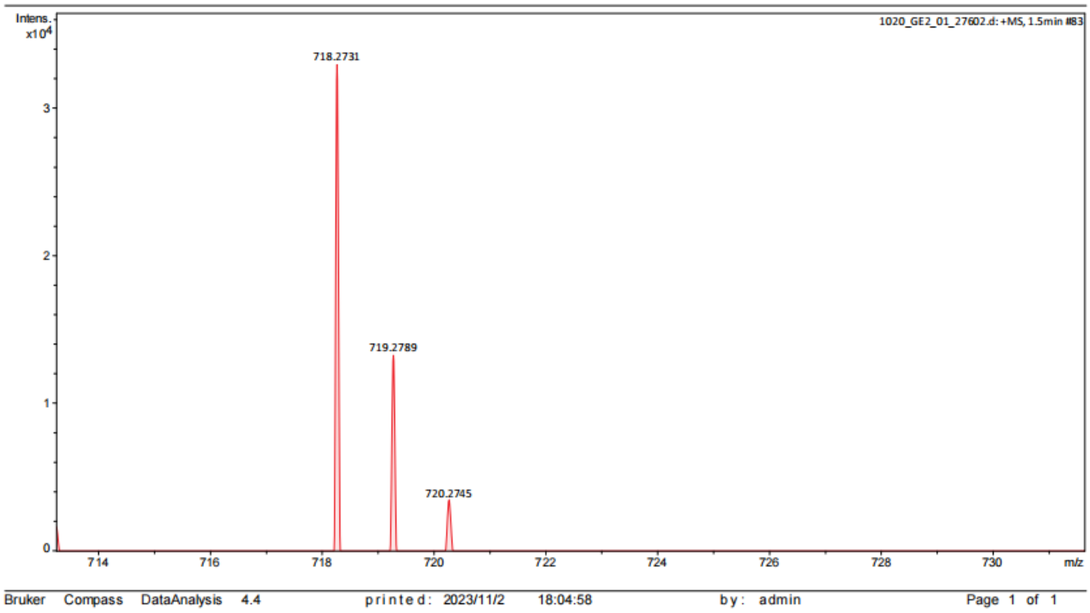

Figure S11-1. ^{1}H NMR spectrum (400 MHz, DMSO-d_6) of compound **4k**

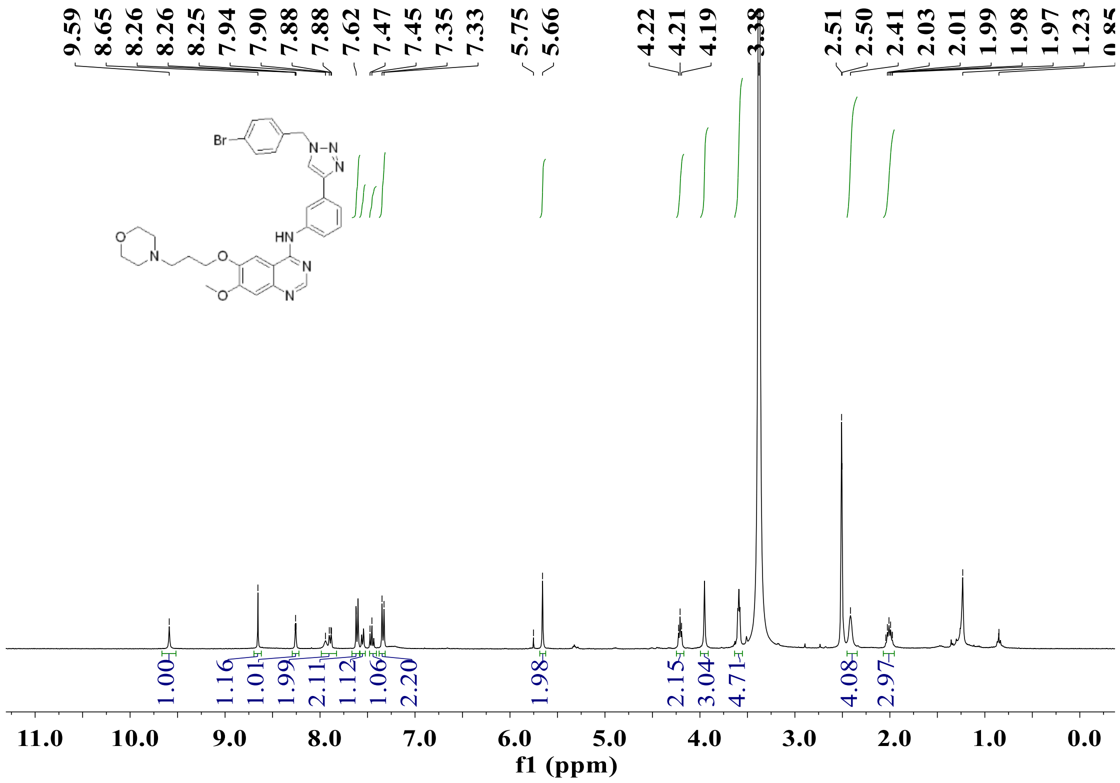

Figure S11-2. ^{13}C NMR spectrum (100 MHz, DMSO-d_6) of compound **4k**

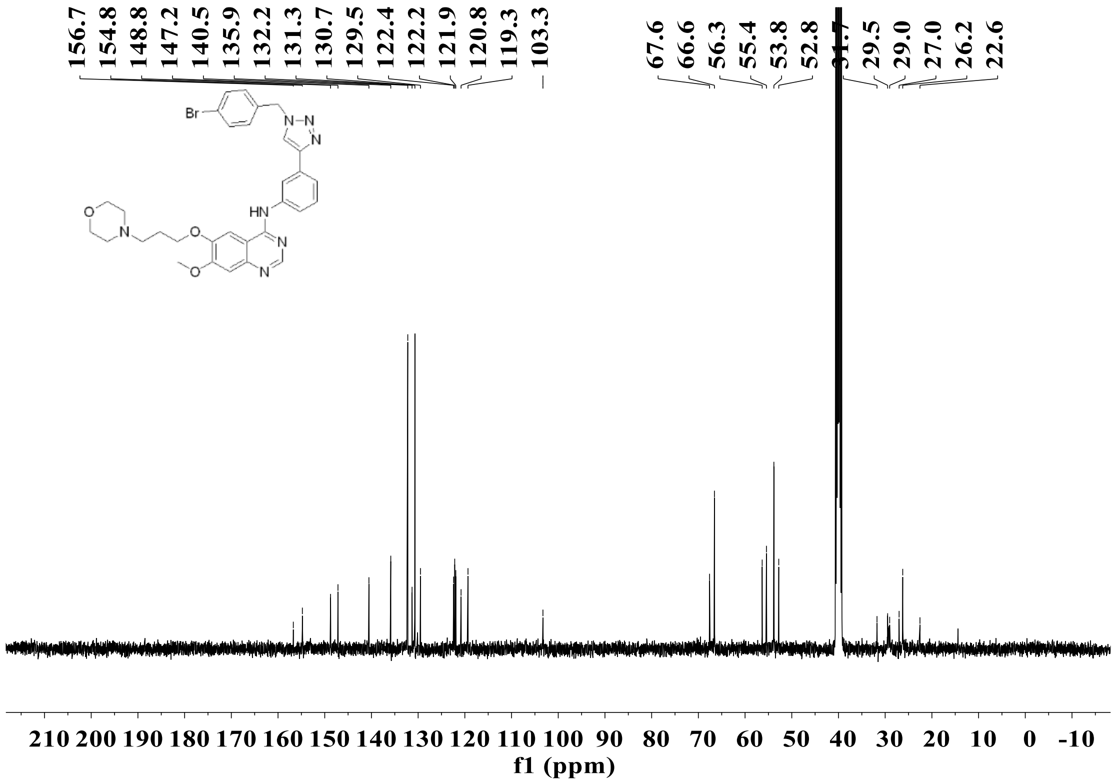

Figure S11-3. HR MS of compound **4k**

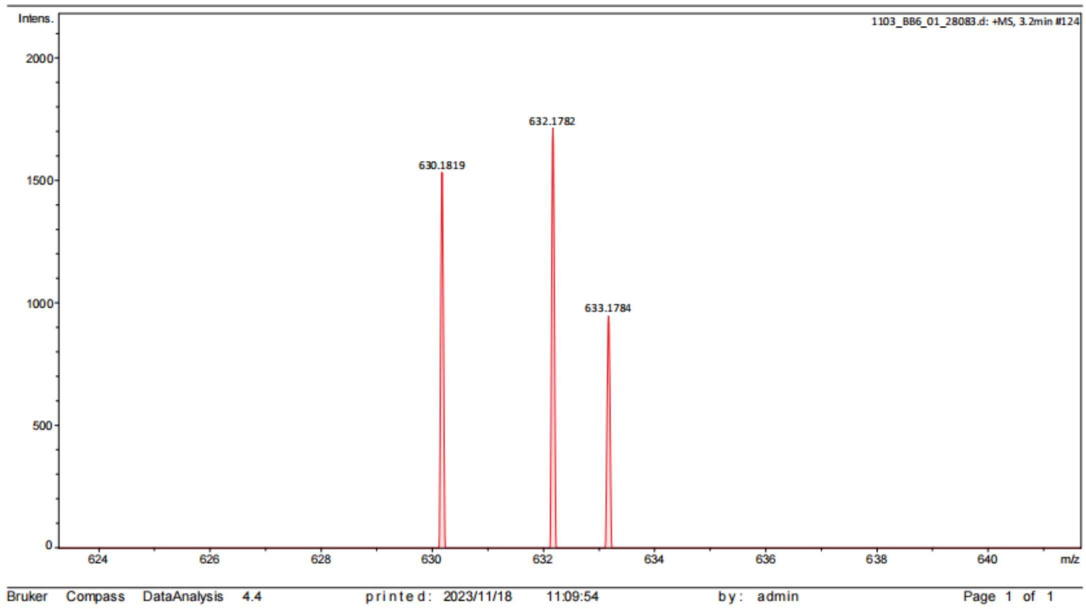

Figure S12-1. ^{1}H NMR spectrum (400 MHz, DMSO-d_6) of compound **4l**

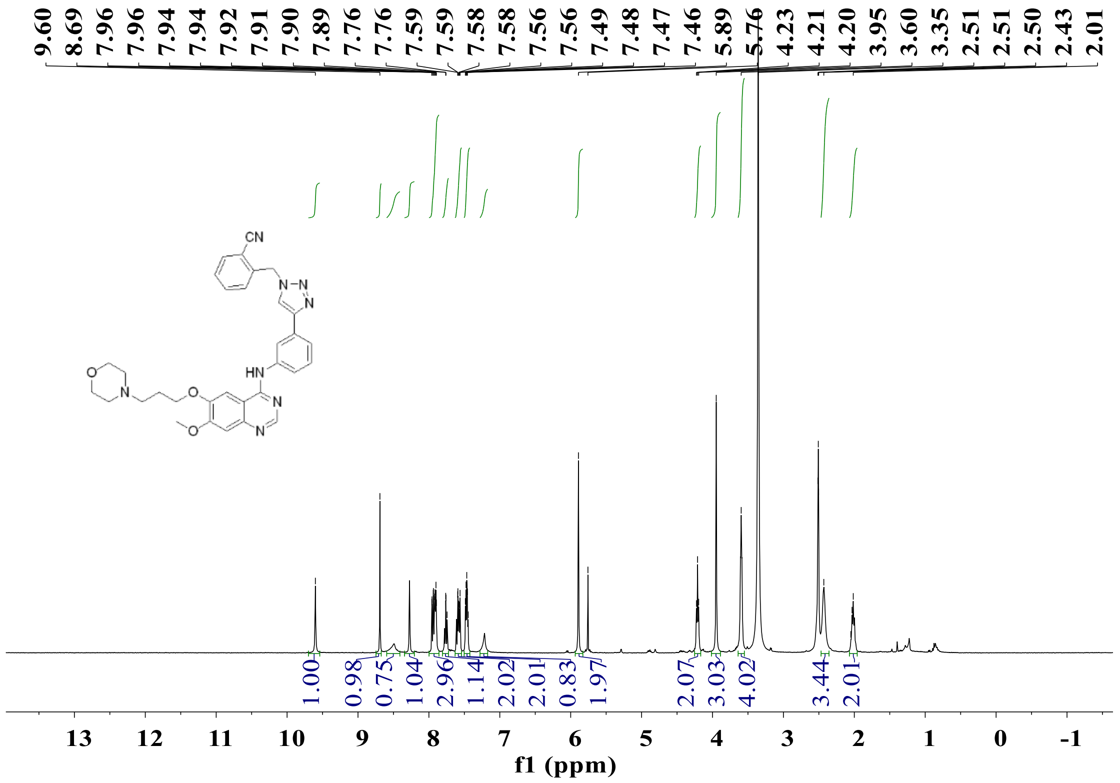

Figure S12-2. ^{13}C NMR spectrum (100 MHz, DMSO-d_6) of compound **4l**

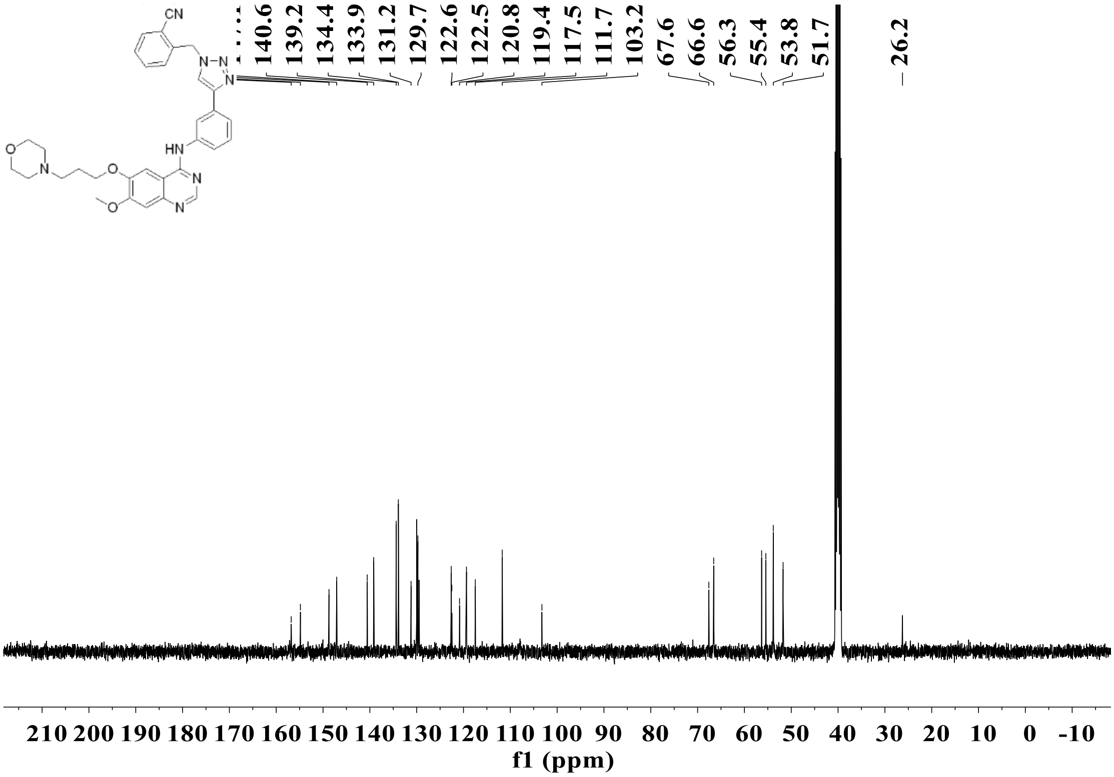

Figure S12-3. HR MS of compound **4l**

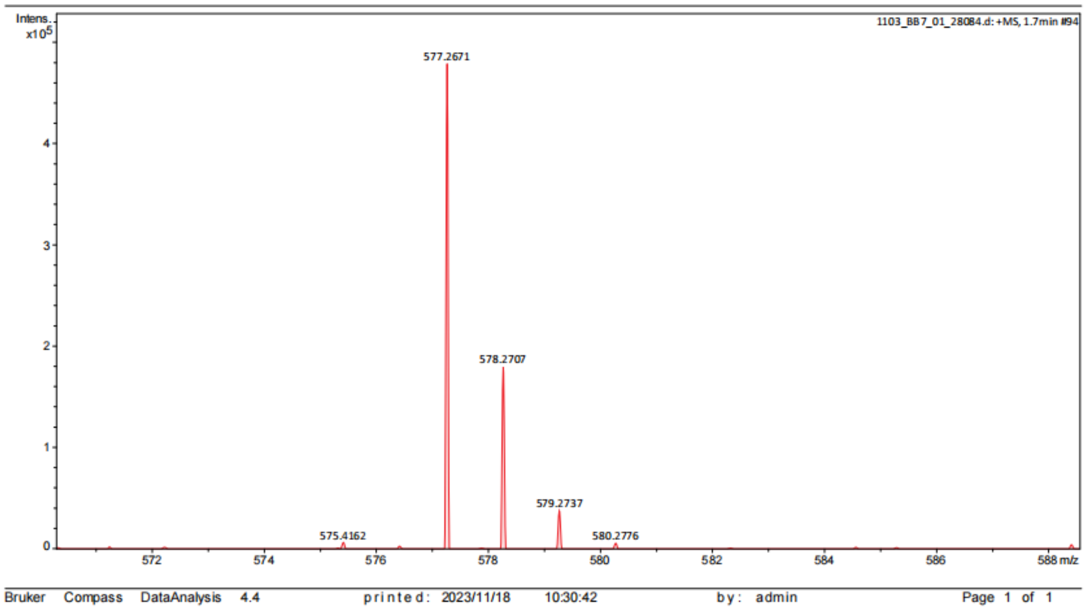

Figure S13-1. ^{1}H NMR spectrum (400 MHz, DMSO-d_6) of compound **4m**

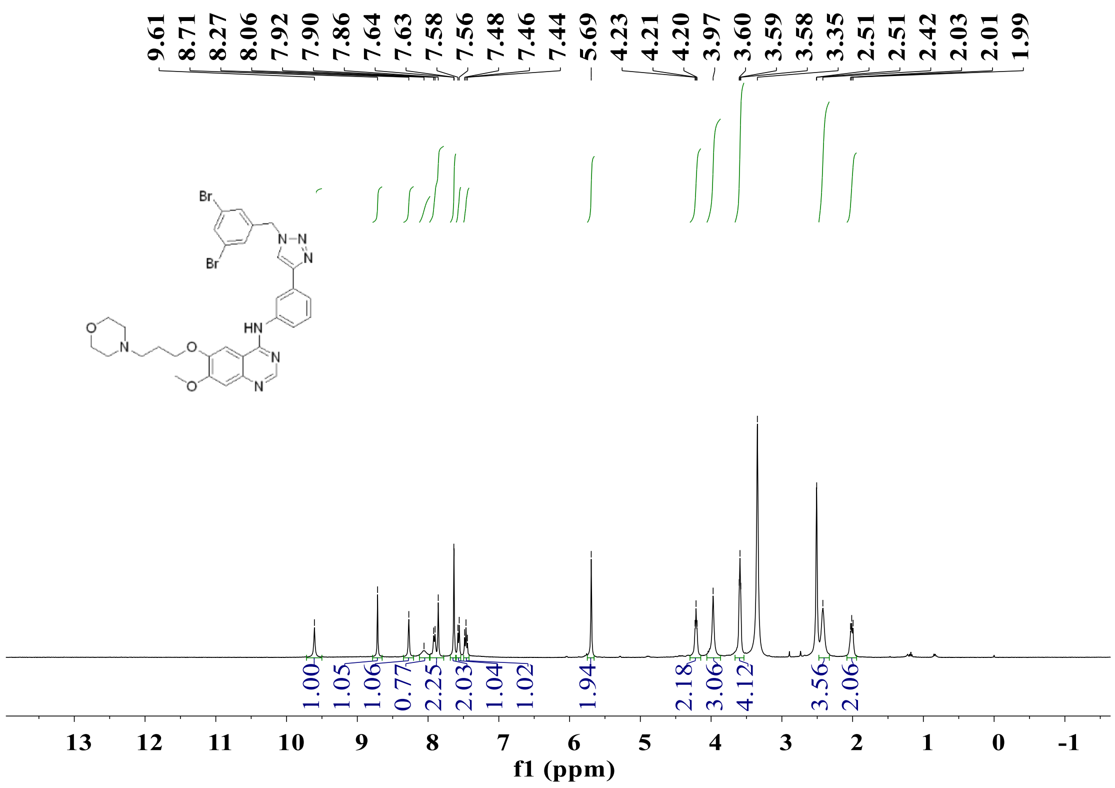

Figure S13-2. ^{13}C NMR spectrum (100 MHz, DMSO-d_6) of compound **4m**

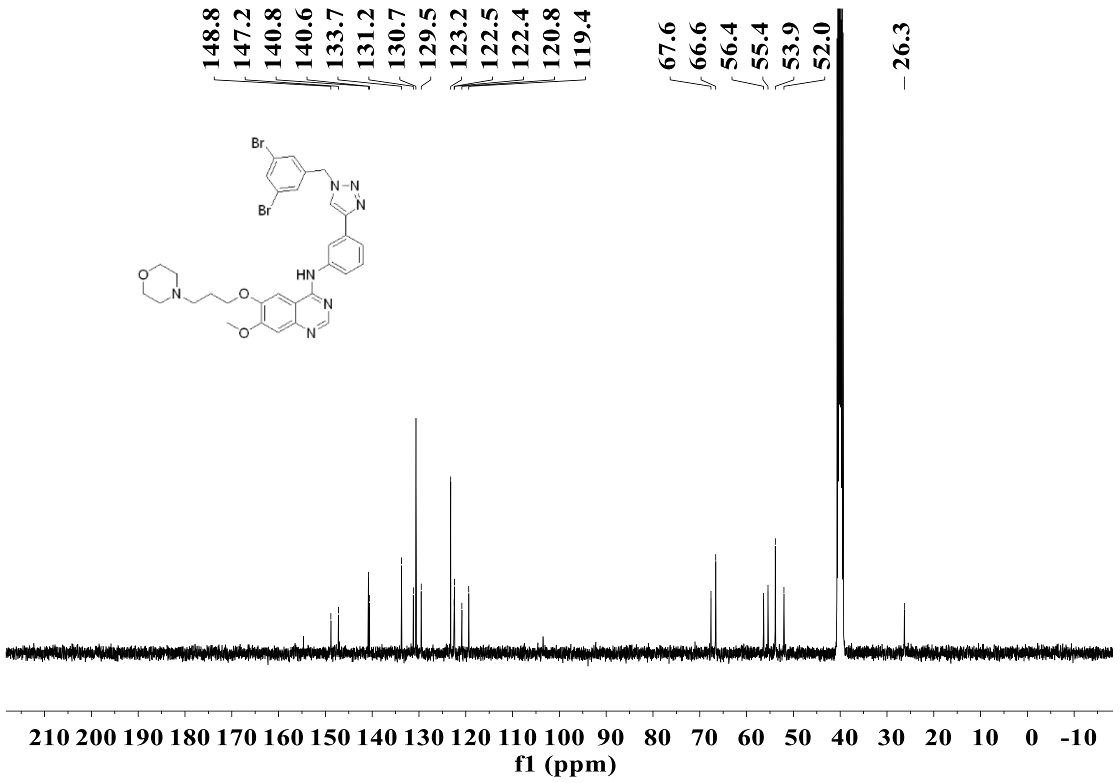

Figure S13-3. HR MS of compound **4m**

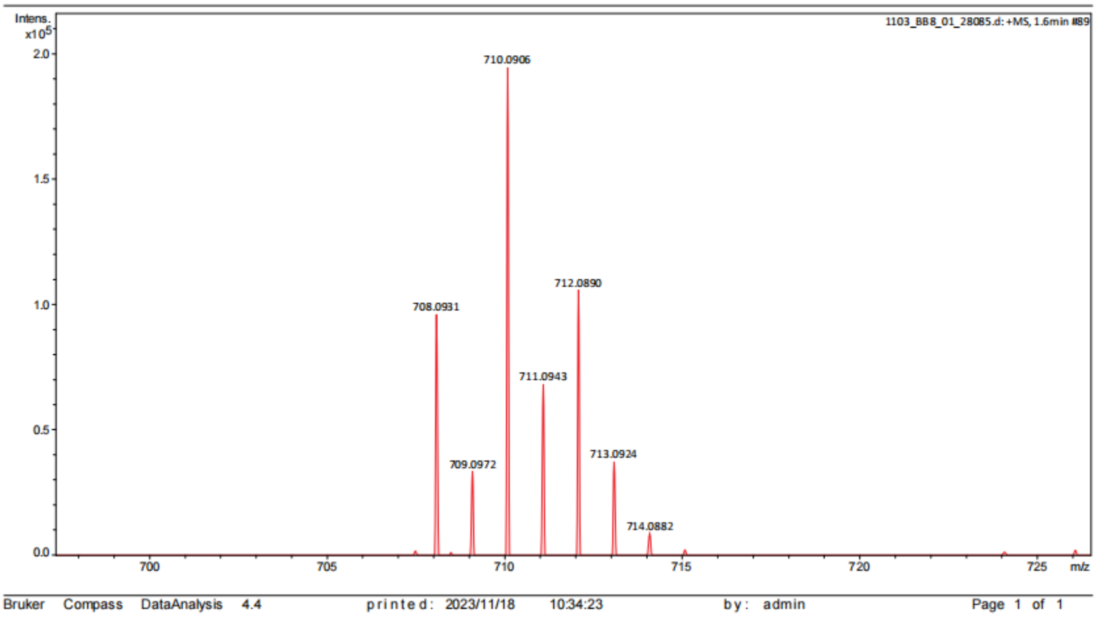

Figure S14-1. ^{1}H NMR spectrum (400 MHz, DMSO-d_6) of compound **4n**

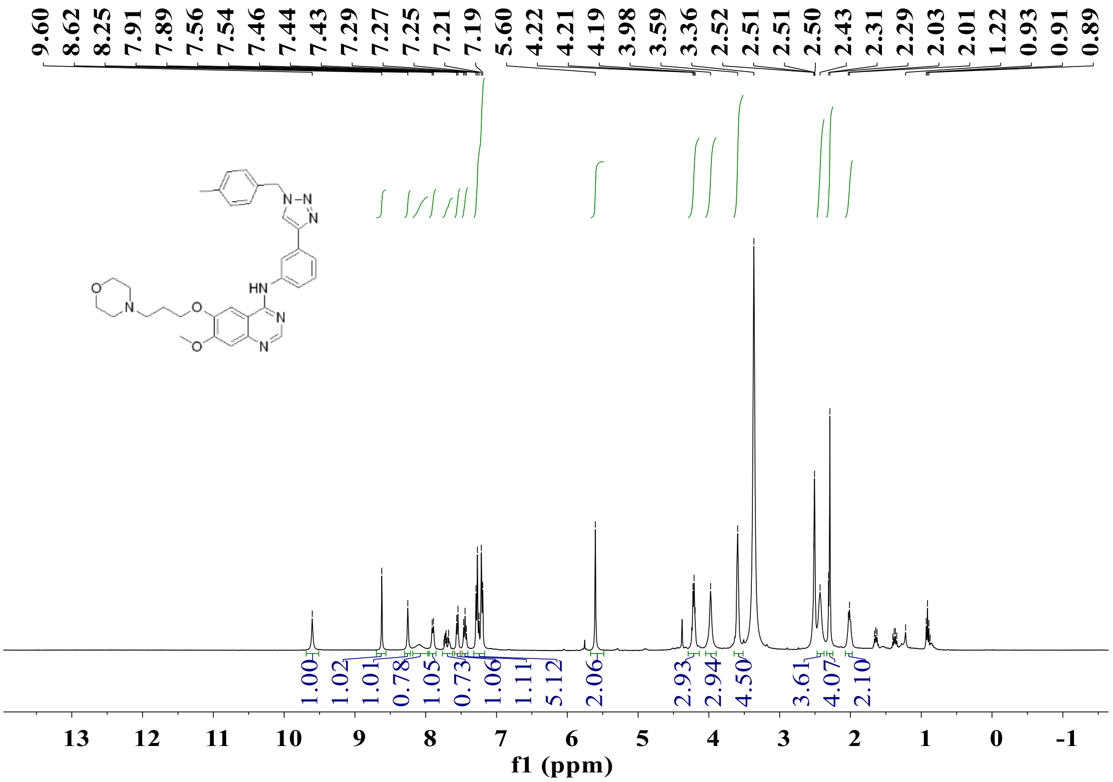

Figure S14-2. ^{13}C NMR spectrum (100 MHz, DMSO-d_6) of compound **4n**

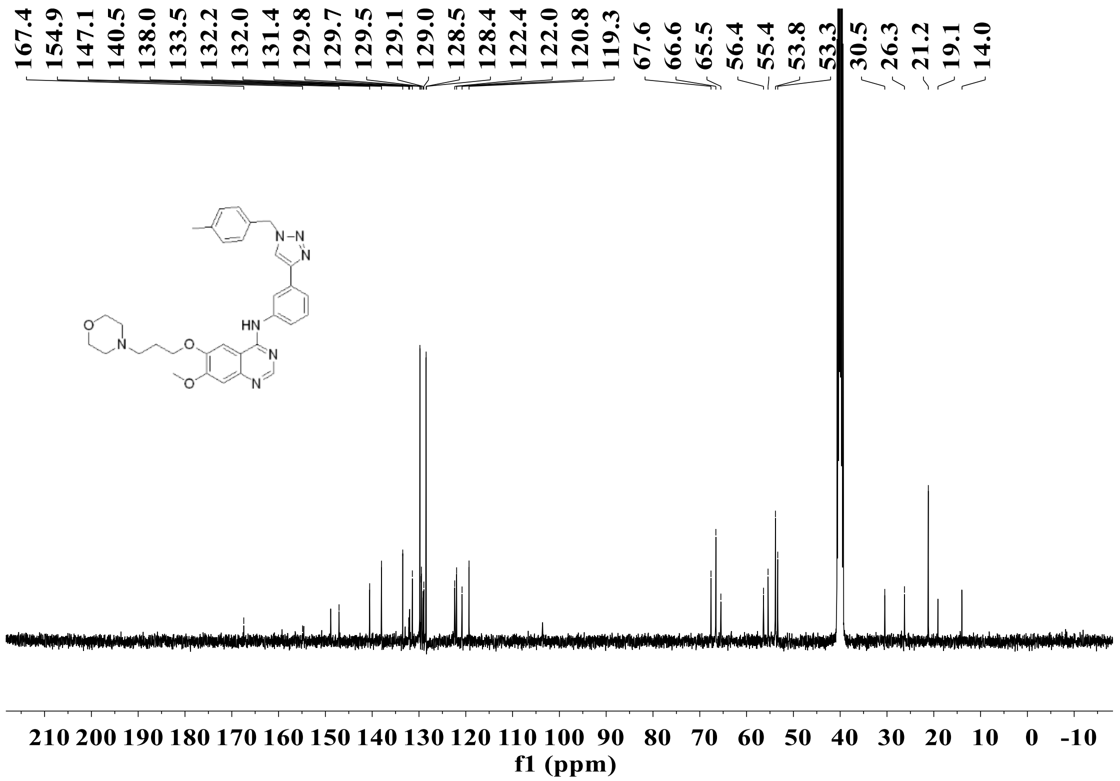

Figure S14-3. HR MS of compound **4n**

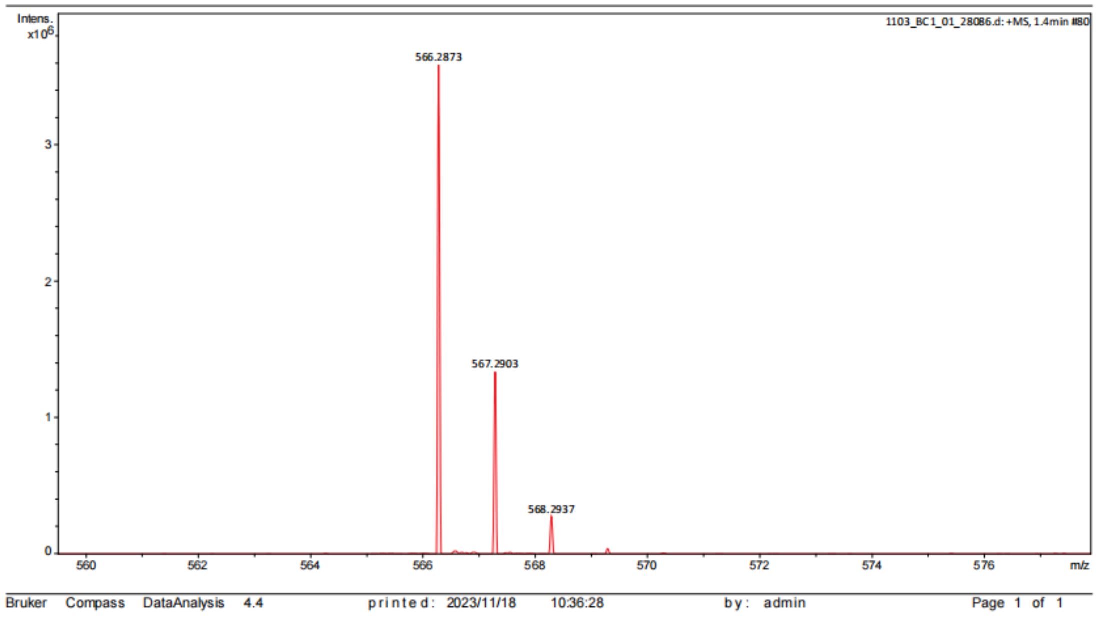

Supplement: Supplementary file 1 [file molecules-29-00837-s001.zip › molecules-2833727-supplementary.pdf]
